# A test of the lateral semicircular canal correlation to head posture, diet and other biological traits in “ungulate” mammals

**DOI:** 10.1038/s41598-020-76757-0

**Published:** 2020-11-11

**Authors:** J. Benoit, L. J. Legendre, A. A. Farke, J. M. Neenan, B. Mennecart, L. Costeur, S. Merigeaud, P. R. Manger

**Affiliations:** 1 Evolutionary Studies Institute (ESI), School of Geosciences, University of the Witwatersrand, Braamfontein, Johannesburg, 2050 South Africa; 2 Jackson School of Geosciences, The University of Texas at Austin, 2275 Speedway Stop C9000, Austin, TX USA; 3 Raymond M. Alf Museum of Paleontology at the Webb Schools, 1175 West Baseline Road, Claremont, CA USA; 4 Oxford University Museum of Natural History, Parks Road, Oxford, OX1 3PW UK; 5 Naturhistorisches Museum Basel, Augustinergasse 2, 4001 Basel, Switzerland; 6 Radiologist Groupe CRP, Clinique du Parc, Castelnau le Lez, France; 7 Tridilogy, Saint Gely du Fesc, France; 8 School of Anatomical Sciences, University of the Witwatersrand, 7 York Road, Parktown, Johannesburg, 2193 South Africa

**Keywords:** Palaeontology, Auditory system

## Abstract

For over a century, researchers have assumed that the plane of the lateral semicircular canal of the inner ear lies parallel to the horizon when the head is at rest, and used this assumption to reconstruct head posture in extinct species. Although this hypothesis has been repeatedly questioned, it has never been tested on a large sample size and at a broad taxonomic scale in mammals. This study presents a comprehensive test of this hypothesis in over one hundred “ungulate” species. Using CT scanning and manual segmentation, the orientation of the skull was reconstructed as if the lateral semicircular canal of the bony labyrinth was aligned horizontally. This reconstructed cranial orientation was statistically compared to the actual head posture of the corresponding species using a dataset of 10,000 photographs and phylogenetic regression analysis. A statistically significant correlation between the reconstructed cranial orientation and head posture is found, although the plane of the lateral semicircular canal departs significantly from horizontal. We thus caution against the use of the lateral semicircular canal as a proxy to infer precisely the horizontal plane on dry skulls and in extinct species. Diet (browsing or grazing) and head-butting behaviour are significantly correlated to the orientation of the lateral semicircular canal, but not to the actual head posture. Head posture and the orientation of the lateral semicircular canal are both strongly correlated with phylogenetic history.

## Introduction

The need for a reliable and reproducible way of orienting dry skulls for cranial measurements has led to a considerable amount of literature suggesting that the plane of the lateral semicircular canal (LSC) of the bony labyrinth (the osseous capsule of the inner ear) is horizontal when the head is held in its “habitual” (i.e. not actively attained) or “alert” positions^1–12^. This is backed by the hypothesis that a horizontal orientation of the LSC would mechanically maximize the recording of rotational and linear head movements made in the horizontal plane by placing the sensory hair cells of the semicircular canal and its associated ampulla perpendicular to the horizontal plane^12–16^. The subsequent use of the orientation of the plane of the LSC as a proxy to infer head posture in fossil vertebrates has grown more popular among paleontologists, as it is being applied to dozens of extinct taxa such as archosaurs, including dinosaurs, and synapsids, including mammals^12,17–29^. This has raised discussion on some crucial paleobiological questions, such as the evolution of bipedalism in ancient hominin^14,18^ and paleodiets. As browsers are expected to hold their head higher than grazers^30^, head posture has been invoked in reconstructing ancient diet in fossil herbivorous species^20,24^. Semi-aquatic species, on the other hand, would hold their head tilted upward^27^ (but see Neenan and Scheyer^31^). In addition, head posture is directly involved in discussions about the origin of endothermy, as blood pressure to perfuse the head, and particularly the brain, directly depends on head posture and thermophysiology (species with low metabolism have a lower blood pressure than species with a high metabolism, and therefore cannot perfuse their brain if their head is held far above their heart)^32^. Head posture may thus be crucial for inferring the evolution of endothermy in birds, mammals, and their respective ancestors, the non-avian dinosaurs, and non-mammalian synapsids^32^. Finally, because of the remodeling of the skull and musculature to accommodate cranial appendages and to absorb shocks, head posture is also central to discussions regarding the practice of display and head-butting^24,29,33^. Head-butting correlates with a hierarchical ranking system and social organisation in herbivores, which makes it a direct proxy of complex behavior in extinct species^34–40^. As such, head posture is relevant to many crucial paleobiological, behavioural, and physiological inferences, and the validity of the use of the LSC orientation as a proxy to reconstruct it requires scrutiny.

Although theoretically sound^13^, and supported by field observations of some reptiles (turtles, crocodiles, and squamates) showing a 0°–5° difference only between the plane of the LSC and the horizontal during “habitual” head posture^12,41–43^, the assumption that the plane of the LSC is horizontal when the head is at rest has been repeatedly challenged in archosaurs and mammals^6,11,43,44^. Most published accounts of head posture in mammals evidence an anterior upward pitching of the LSC averaging 20°–30° to the horizontal (e.g. in humans) when the head is at rest or in alert posture^6,11,12,30,43,45^. In birds, the orientation of the plane of the LSC in alert posture varies between − 15° and 50° around the horizontal^8,43^. All of these studies used different methodological approaches to quantify head posture (e.g. by keeping animals on a leash or distracted, using field observation of wild animals, using photographs from open access banks, using pictures from the literature) and access the orientation of the LSC (e.g. using X-ray radiography, CT scanning, sawed skulls or dissection), thus making their results difficult to compare^12,43^. In addition, these studies were made on a small number of individuals, (domestic animals and rodents usually) even though studies in humans have shown that the orientation of the plane of the LSC can vary a lot within species (e.g. Caix and Outrequin, 1979). As such, though generally accepted, robust evidence in support of the hypothesis that the orientation of the plane of the LSC is horizontal when the animal is at rest, and can, therefore, be used to reconstruct head posture in extinct species, is still pending.

This study implements a large scale and methodologically homogenous critical assessment of the question using a statistical approach in modern “ungulates” (Perissodactyla, Artiodactyla, and Paenungulata). The aim is to document the actual, neutral head posture in life of modern species (using field observations) in order to compare this to the head posture inferred from LSC orientation in a dry skull (using CT scanning). This will enable us to address if the orientation of the plane of the LSC is a good proxy to reconstruct the head posture of extinct species, in order to ultimately make future paleobiological reconstructions more reliable. We will also test if some variables such as diet, body size, habitat, and head-butting are significantly correlated to head posture and/or LSC orientation as is usually believed^24,30,45,47^.

## Materials and methods

### Sampling

As the inclusion of a statistically-significant number of taxa was essential to this study, we chose to focus primarily on ungulate-grade mammals (i.e. Paenungulata, Artiodactyla, Perissodactyla, and Tubulidentata). Ungulates are more abundant than carnivores or primates in zoos, easily identifiable, and well represented in institutional dry skull collections. They display a wide array of body sizes, a greater variety of documented head postures^47^, a wider range of expected inner ear orientations (as hypothesized from the inclination of the snout compared to that of the brain-case^48^), and more varied degrees of adaptation to head-butting^34^ than any other mammalian group. Moreover, they are the ideal target group to address if diet (browsing v. grazing) plays a significant role in the orientation of the LSC, as previously suggested in the literature^20,24^. Finally, they usually display an elongated snout, which makes it easier to compare the orientation of the head in live animals to that of the corresponding dry skulls.

### Head posture in live animals

**Figure 1:**
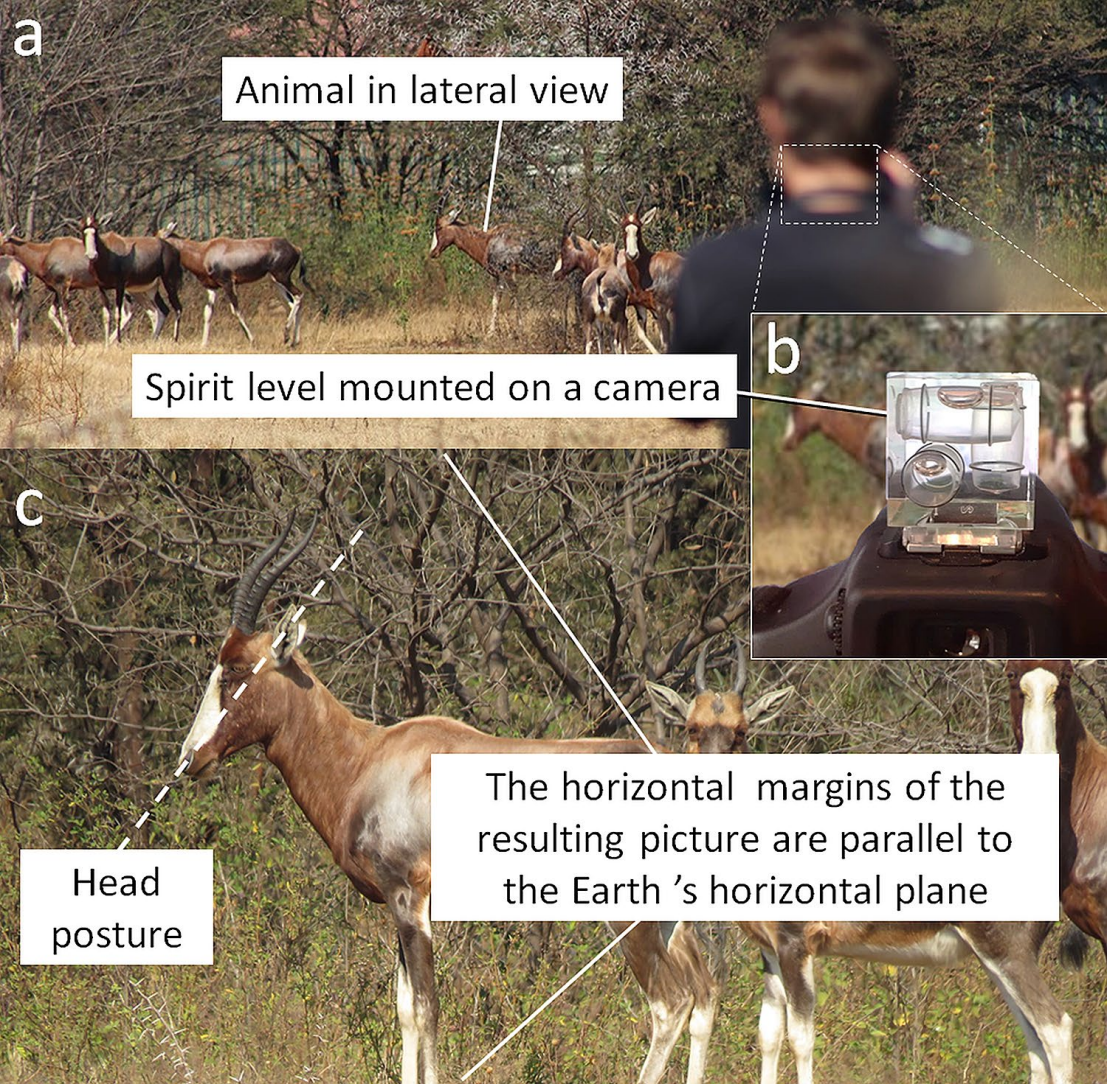
Protocol to photograph animal head posture. (**a**) An animal stands or slowly walks The camera is positioned to photograph the animal in lateral view. (**b**) A spirit-level mounted on top of the camera ensures that the picture is captured when the camera is held horizontally. (**c**) The borders of the resulting picture are parallel to the horizontal plane (which enables measurement of neutral head posture).

Head posture was documented by taking pictures of zoo animals in lateral view using a camera equipped with a spirit level (Fig. 1) to ensure that pictures were taken as close to the horizontal plane as possible. The animals were photographed in 2018 and 2019 at the National Zoological Garden, Pretoria (South Africa), Johannesburg Zoo (South Africa), Montecasino Bird Garden, Fourways (South Africa), Lory Park Animal and Owl Sanctuary, Midrand (South Africa), Ménagerie du Jardin des Plantes, Paris (France), Parc Zoologique de Paris (France), Prague Zoo (Czech Republic), Chester Zoo (United Kingdom), Zoologischer Garten Berlin (Germany), Tierpark Berlin (Germany), and Zooparc of Beauval (France). The saiga antelope pictures were kindly provided by K.H. Vogel. The dataset represents about 10,000 pictures documenting the head posture of 129 species and is available here: https://osf.io/4vpnj/?view_only=3dc987012fcd44a6a64ad7d8949ec01f (https://doi.org/10.17605/OSF.IO/4VPNJ). The pictures were taken from outside the enclosures to avoid interaction with the animals. It was essential for this study that the animals remain calm and act naturally, so their environment was not disturbed, and the animals were not put on leash or isolated. As such, individual identification was not possible. Representatives of both sexes are mixed in the dataset as sexes could not always be determined. The typical photography set up is illustrated in Fig. 1.

To ensure that the photographed head postures were comparable between individuals and species, all pictures were taken by one of the authors only (J.B., except for the saigas, which were taken by Alexander Sliwa from the Kölner Zoo). The pictures used for this study were selected to reflect as closely as possible what will hereafter be referred to as the “neutral” head posture. The neutral posture of an “ungulate” is here defined as the angle between the main axis of the head and the horizontal when an animal’s head remains still, its attention is not attracted by a moving or immobile target, and it is not foraging, drinking, or performing any other identifiable activity involving head movements (e.g. sniffing). The animal can be standing or lying down. “Neutral” head posture differs from head posture “at rest” as it encompasses ruminating animals and individuals slowly walking with their head steady (not pitching up and down while moving). Alert postures^49^ were included only if the animal's attention was not directed toward an identifiable direction, and were avoided as much as possible. That is why this study focuses on zoo animals, which are accustomed to human presence. For consistency and to enable comparisons, pictures of semiaquatic “ungulates” (e.g. hippos) were taken when the animal’s head was not immersed so that their head posture was not influenced by buoyancy.

**Figure 2:**
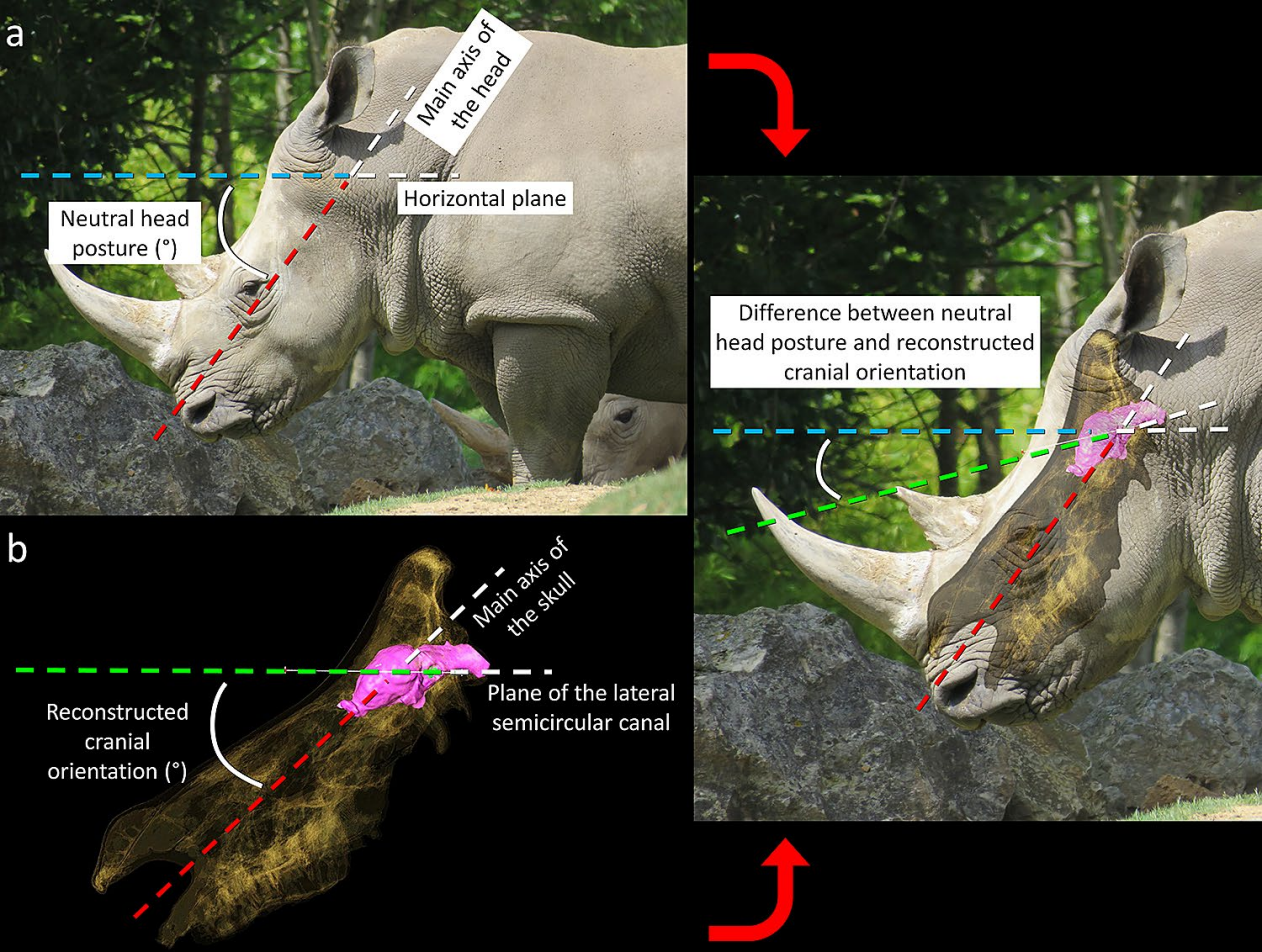
Measurement protocols illustrated on a white rhinoceros (*Ceratotherium simum*). (**a**) Neutral head posture measured from the photographs (see Fig. 1). (**b**) Reconstructed cranial orientation from the CT-scans. (**c**) A combination of the two measurements that illustrates how the orientation of the plane of the laterals semicircular canal compares to the horizontal.

The orientation of the head compared to the horizontal plane was measured by J.B. using ImageJ as the angle between the horizontal border of the picture (horizontality of which was ensured by the use of a spirit level on the camera, Fig. 1) and the main axis of the head (traced as the axis running from just above the upper lip to the middle of the occiput on the back of the head) in strict lateral view (Fig. 2a). An average neutral head posture was then calculated for each species (Table 1). The intraspecific standard deviation (measurement error) for neutral head posture is ± 1.6°.

**Table 1:** Dataset used for the statistic analyses.

Species	Order	Sub-order	Average reconstructed cranial orientation (in °)	Average neutral head posture (in °)	Body mass (in kg)	Diet: browser/grazer/mixed/other	Frontal head to head butting: yes/no	Habitat: open/closed/mixed/rocky/semi-aquatic
*Elephas maximus*	Afrotheria	Paenungulata	?	39 (n = 105)	2720	Mixed	Yes	Mixed
*Heterohyrax brucei*	Afrotheria	Paenungulata	?	32 (n = 95)	2	Browser	No	Rocky
*Loxodonta africana*	Afrotheria	Paenungulata	28 (n = 3)	48 (n = 103)	4000	Mixed	Yes	Open
*Procavia capensis*	Afrotheria	Paenungulata	21 (n = 4)	19 (n = 121)	4	Grazer	No	Rocky
*Orycteropus afer*	Afrotheria	Tubulidentata	41 (n = 2)	42 (n = 37)	70	Other	No	Open
*Hexaprotodon liberiensis*	Artiodactyla	Hippopotamidae	27 (n = 2)	50 (n = 27)	262	Browser	No	Semi-aquatic
*Hippopotamus amphibius*	Artiodactyla	Hippopotamidae	20 (n = 3)	59 (n = 74)	2300	Grazer	No	Semi-aquatic
*Addax nasomaculatus*	Artiodactyla	Ruminantia	45 (n = 1)	48 (n = 75)	93	Grazer	Yes	Open
*Aepyceros melampus*	Artiodactyla	Ruminantia	53 (n = 2)	24 (n = 41)	58	Mixed	Yes	Open
*Alcelaphus buselaphus*	Artiodactyla	Ruminantia	68 (n = 8)	55 (n = 67)	185	Grazer	Yes	Open
*Alces alces*	Artiodactyla	Ruminantia	44 (n = 2)	33 (n = 28)	543	Browser	Yes	Closed
*Ammotragus lervia*	Artiodactyla	Ruminantia	51 (n = 1)	43 (n = 71)	103	Mixed	Yes	Rocky
*Antidorcas marsupialis*	Artiodactyla	Ruminantia	42 (n = 3)	38 (n = 61)	43	Mixed	Yes	Open
*Antilocapra americana*	Artiodactyla	Ruminantia	42 (n = 1)	36 (n = 140)	55	Browser	Yes	Open
*Antilope cervicapra*	Artiodactyla	Ruminantia	35 (n = 3)	23 (n = 121)	37	Grazer	Yes	Open
*Axis axis*	Artiodactyla	Ruminantia	46 (n = 1)	27 (n = 122)	78	Grazer	Yes	Open
*Axis kuhlii*	Artiodactyla	Ruminantia	?	24 (n = 8)	36	Grazer	?	Open
*Axis porcinus*	Artiodactyla	Ruminantia	35 (n = 1)	28 (n = 117)	43	Grazer	?	Open
*Babyrousa babyrussa*	Artiodactyla	Ruminantia	?	38 (n = 13)	90	Other	No	Closed
*Babyrousa celebensis*	Artiodactyla	Ruminantia	?	29 (n = 18)	90	Other	No	Closed
*Bison bison*	Artiodactyla	Ruminantia	70 (n = 3)	51 (n = 119)	613	Grazer	Yes	Mixed
*Bison bonasus*	Artiodactyla	Ruminantia	?	49 (n = 44)	610	Mixed	Yes	Mixed
*Bos gaurus*	Artiodactyla	Ruminantia	?	33 (n = 37)	825	Mixed	No	Closed
*Bos grunniens*	Artiodactyla	Ruminantia	?	49 (n = 13)	775	Grazer	Yes	Open
*Bos javanicus*	Artiodactyla	Ruminantia	32 (n = 1)	29 (n = 71)	650	Grazer	?	Closed
*Bos taurus*	Artiodactyla	Ruminantia	36 (n = 2)	39 (n = 89)	650	Grazer	Yes	Open
*Boselaphus tragocamelus*	Artiodactyla	Ruminantia	36 (n = 4)	28 (n = 127)	205	Mixed	No	Open
*Bubalus bubalis*	Artiodactyla	Ruminantia	?	31 (n = 112)	771	Mixed	Yes	Closed
*Bubalus depressicornis*	Artiodactyla	Ruminantia	51 (n = 3)	30 (n = 81)	250	Mixed	?	Closed
*Bubalus mindorensis*	Artiodactyla	Ruminantia	49 (n = 1)	?	240	Grazer	No	Closed
*Budorcas taxicolor*	Artiodactyla	Ruminantia	54 (n = 2)	46 (n = 121)	300	Mixed	Yes	Closed
*Capra caucasica*	Artiodactyla	Ruminantia	?	36 (n = 60)	96	Mixed	Yes	Rocky
*Capra falconeri*	Artiodactyla	Ruminantia	52 (n = 1)	30 (n = 48)	71	Mixed	Yes	Closed
*Capra hircus*	Artiodactyla	Ruminantia	45 (n = 4)	32 (n = 164)	80	Mixed	Yes	Open
*Capra ibex*	Artiodactyla	Ruminantia	56 (n = 3)	25 (n = 72)	99	Grazer	Yes	Rocky
*Capreolus capreolus*	Artiodactyla	Ruminantia	43 (n = 1)	28 (n = 92)	28	Mixed	No	Mixed
*Cephalophus dorsalis*	Artiodactyla	Ruminantia	40 (n = 3)	?	20	Browser	No	Closed
*Cephalophus leucogaster*	Artiodactyla	Ruminantia	38 (n = 4)	?	18	Browser	No	Closed
*Cephalophus natalensis*	Artiodactyla	Ruminantia	30 (n = 3)	25 (n = 68)	13	Browser	No	Closed
*Cephalophus niger*	Artiodactyla	Ruminantia	36 (n = 3)	15 (n = 100)	20	Browser	No	Closed
*Cephalophus nigrifrons*	Artiodactyla	Ruminantia	31 (n = 5)	?	17	Browser	No	Closed
*Cephalophus sylvicultor*	Artiodactyla	Ruminantia	43 (n = 3)	31 (n = 148)	63	Browser	No	Closed
*Cervus albirostris*	Artiodactyla	Ruminantia	?	30 (n = 61)	165	Grazer	Yes	Mixed
*Cervus elaphus*	Artiodactyla	Ruminantia	41 (n = 5)	22 (n = 186)	128	Mixed	Yes	Mixed
*Cervus nippon*	Artiodactyla	Ruminantia	35 (n = 1)	19 (n = 20)	95	Mixed	Yes	Closed
*Connochaetes gnou*	Artiodactyla	Ruminantia	?	62 (n = 14)	134	Mixed	Yes	Open
*Connochaetes taurinus*	Artiodactyla	Ruminantia	68 (n = 4)	60 (n = 74)	213	Grazer	Yes	Open
*Dama dama*	Artiodactyla	Ruminantia	48 (n = 3)	25 (n = 69)	35	Grazer	Yes	Mixed
*Damaliscus lunatus*	Artiodactyla	Ruminantia	55 (n = 6)	?	155	Grazer	Yes	Open
*Damaliscus pygargus*	Artiodactyla	Ruminantia	54 (n = 2)	50 (n = 81)	71	Grazer	Yes	Open
*Elaphodus cephalophus*	Artiodactyla	Ruminantia	27 (n = 1)	33 (n = 15)	22	Mixed	No	Closed
*Elaphurus davidianus*	Artiodactyla	Ruminantia	46 (n = 2)	38 (n = 73)	183	Grazer	No	Open
*Gazella dorcas*	Artiodactyla	Ruminantia	30 (n = 3)	?	15	Browser	No	Open
*Gazella spekei*	Artiodactyla	Ruminantia	?	28 (n = 154)	20	Mixed	?	Open
*Gazella subgutturosa*	Artiodactyla	Ruminantia	42 (n = 2)	?	28	Mixed	No	Open
*Gazella thomsonii*	Artiodactyla	Ruminantia	46 (n = 3)	?	21	Grazer	Yes	Open
*Giraffa camelopardalis*	Artiodactyla	Ruminantia	32 (n = 5)	24 (n = 187)	1317	Browser	No	Open
*Hemitragus jemlahicus*	Artiodactyla	Ruminantia	59 (n = 2)	40 (n = 79)	75	Mixed	Yes	Rocky
*Hippocamelus sp.*	Artiodactyla	Ruminantia	56 (n = 1)	?	55	Browser	?	Mixed
*Hippotragus equinus*	Artiodactyla	Ruminantia	46 (n = 1)	36 (n = 78)	262	Grazer	Yes	Open
*Hippotragus niger*	Artiodactyla	Ruminantia	44 (n = 2)	40 (n = 140)	228	Grazer	Yes	Open
*Hydropotes inermis*	Artiodactyla	Ruminantia	?	28 (n = 5)	12	Mixed	No	Open
*Kobus ellipsiprymnus*	Artiodactyla	Ruminantia	42 (n = 4)	31 (n = 76)	211	Grazer	Yes	Mixed
*Kobus leche*	Artiodactyla	Ruminantia	46 (n = 2)	27 (n = 59)	91	Grazer	Yes	Open
*Kobus megaceros*	Artiodactyla	Ruminantia	?	17 (n = 68)	90	Grazer	Yes	Open
*Kobus vardoni*	Artiodactyla	Ruminantia	39 (n = 2)	?	66	Grazer	Yes	Open
*Litocranius walleri*	Artiodactyla	Ruminantia	33 (n = 3)	14 (n = 181)	41	Browser	No	Open
*Madoqua sp.*	Artiodactyla	Ruminantia	25 (n = 3)	24 (n = 97)	6	Browser	No	Open
*Mazama americana*	Artiodactyla	Ruminantia	35 (n = 1)	?	17	Mixed	No	Closed
*Moschus moschiferus*	Artiodactyla	Ruminantia	25 (n = 1)	32 (n = 16)	12	Mixed	No	Closed
*Muntiacus reevesi*	Artiodactyla	Ruminantia	43 (n = 2)	30 (n = 78)	21	Mixed	No	Closed
*Naemorhedus crispus*	Artiodactyla	Ruminantia	50 (n = 1)	?	38	Mixed	No	Rocky
*Naemorhedus goral*	Artiodactyla	Ruminantia	41 (n = 2)	38 (n = 33)	29	Mixed	No	Rocky
*Naemorhedus griseus*	Artiodactyla	Ruminantia	?	38 (n = 10)	29	Mixed	?	Rocky
*Naemorhedus sumatraensis*	Artiodactyla	Ruminantia	47 (n = 3)	?	29	Mixed	?	Closed
*Nanger dama*	Artiodactyla	Ruminantia	?	23 (n = 41)	46	Mixed	Yes	Open
*Nanger granti*	Artiodactyla	Ruminantia	39 (n = 4)	?	56	Mixed	Yes	Open
*Nanger soemmeringii*	Artiodactyla	Ruminantia	?	25 (n = 108)	42	Mixed	Yes	Open
*Neotragus batesi*	Artiodactyla	Ruminantia	28 (n = 3)	?	4	Browser	No	Closed
*Nesotragus moschatus*	Artiodactyla	Ruminantia	26 (n = 2)	?	4	Browser	No	Closed
*Nilgiritragus hylocrius*	Artiodactyla	Ruminantia	61 (n = 2)	?	90	Grazer	Yes	Rocky
*Odocoileus virginianus*	Artiodactyla	Ruminantia	?	19 (n = 22)	79	Mixed	Yes	Closed
*Okapia johnstoni*	Artiodactyla	Ruminantia	40 (n = 2)	34 (n = 193)	283	Browser	No	Closed
*Oreamnos americanus*	Artiodactyla	Ruminantia	50 (n = 3)	52 (n = 52)	91	Grazer	No	Rocky
*Oreotragus oreotragus*	Artiodactyla	Ruminantia	34 (n = 3)	15 (n = 262)	14	Browser	Yes	Rocky
*Oryx beisa*	Artiodactyla	Ruminantia	?	48 (n = 48)	163	Mixed	Yes	Open
*Oryx dammah*	Artiodactyla	Ruminantia	46 (n = 1)	44 (n = 87)	137	Grazer	Yes	Open
*Oryx gazella*	Artiodactyla	Ruminantia	49 (n = 2)	45 (n = 60)	163	Grazer	Yes	Open
*Oryx leucoryx*	Artiodactyla	Ruminantia	?	43 (n = 60)	75	Grazer	Yes	Open
*Ourebia ourebi*	Artiodactyla	Ruminantia	31 (n = 3)	?	12	Grazer	No	Open
*Ovibos moschatus*	Artiodactyla	Ruminantia	42 (n = 2)	46 (n = 21)	475	Mixed	Yes	Open
*Ovis ammon*	Artiodactyla	Ruminantia	36 (n = 1)	23 (n = 53)	163	Mixed	Yes	Rocky
*Ovis aries*	Artiodactyla	Ruminantia	37 (n = 2)	22 (n = 104)	110	Mixed	Yes	Open
*Ovis canadensis*	Artiodactyla	Ruminantia	55 (n = 2)	?	98	Browser	Yes	Rocky
*Ovis orientalis*	Artiodactyla	Ruminantia	31 (n = 1)	28 (n = 11)	48	Grazer	Yes	Rocky
*Panolia eldii*	Artiodactyla	Ruminantia	?	20 (n = 60)	93	Grazer	?	Open
*Pantholops hodgsonii*	Artiodactyla	Ruminantia	46 (n = 3)	?	33	Grazer	Yes	Open
*Pelea capreolus*	Artiodactyla	Ruminantia	45 (n = 3)	?	25	Mixed	No	Open
*Philantomba monticola*	Artiodactyla	Ruminantia	16 (n = 1)	26 (n = 23)	7	Browser	No	Closed
*Procapra gutturosa*	Artiodactyla	Ruminantia	38 (n = 3)	?	30	Grazer	No	Open
*Pseudois nayaur*	Artiodactyla	Ruminantia	65 (n = 1)	28 (n = 73)	55	Grazer	Yes	Rocky
*Pudu puda*	Artiodactyla	Ruminantia	22 (n = 1)	27 (n = 30)	10	Browser	No	Closed
*Rangifer tarandus*	Artiodactyla	Ruminantia	45 (n = 2)	35 (n = 189)	99	Grazer	Yes	Mixed
*Raphicerus campestris*	Artiodactyla	Ruminantia	28 (n = 5)	28 (n = 9)	11	Mixed	No	Open
*Redunca arundinum*	Artiodactyla	Ruminantia	34 (n = 2)	23 (n = 128)	73	Grazer	Yes	Open
*Redunca fulvorufula*	Artiodactyla	Ruminantia	39 (n = 3)	23 (n = 33)	29	Grazer	Yes	Open
*Rucervus duvauceli*	Artiodactyla	Ruminantia	?	23 (n = 17)	180	Grazer	?	Open
*Rupicapra rupicapra*	Artiodactyla	Ruminantia	42 (n = 2)	36 (n = 84)	37	Grazer	Yes	Rocky
*Rusa alfredi*	Artiodactyla	Ruminantia	?	24 (n = 10)	53	Mixed	?	Closed
*Rusa timorensis*	Artiodactyla	Ruminantia	47 (n = 1)	29 (n = 32)	117	Grazer	?	Open
*Rusa unicolor*	Artiodactyla	Ruminantia	38 (n = 1)	21 (n = 17)	273	Mixed	Yes	Mixed
*Saiga tatarica*	Artiodactyla	Ruminantia	43 (n = 2)	38 (n = 2)	48	Grazer	Yes	Open
*Sylvicapra grimmia*	Artiodactyla	Ruminantia	30 (n = 4)	?	19	Browser	No	Mixed
*Syncerus caffer caffer*	Artiodactyla	Ruminantia	51 (n = 2)	27 (n = 70)	648	Grazer	Yes	Open
*Syncerus caffer nanus*	Artiodactyla	Ruminantia	?	24 (n = 72)	320	Mixed	No	Closed
*Taurotragus derbianus*	Artiodactyla	Ruminantia	?	27 (n = 55)	700	Browser	Yes	Closed
*Taurotragus oryx*	Artiodactyla	Ruminantia	49 (n = 4)	30 (n = 53)	470	Browser	Yes	Mixed
*Tetracerus quadricornis*	Artiodactyla	Ruminantia	31 (n = 3)	?	17	Browser	No	Mixed
*Tragelaphus angasii*	Artiodactyla	Ruminantia	29 (n = 3)	22 (n = 76)	78	Mixed	Yes	Closed
*Tragelaphus euryceros*	Artiodactyla	Ruminantia	25 (n = 1)	26 (n = 197)	330	Browser	No	Closed
*Tragelaphus imberbis*	Artiodactyla	Ruminantia	46 (n = 1)	21 (n = 252)	82	Browser	Yes	Closed
*Tragelaphus scriptus*	Artiodactyla	Ruminantia	26 (n = 3)	25 (n = 59)	53	Browser	Yes	Closed
*Tragelaphus spekei*	Artiodactyla	Ruminantia	35 (n = 1)	20 (n = 125)	98	Mixed	Yes	Closed
*Tragelaphus strepsiceros*	Artiodactyla	Ruminantia	41 (n = 4)	23 (n = 78)	214	Browser	Yes	Mixed
*Tragulus javanicus*	Artiodactyla	Ruminantia	33 (n = 2)	15 (n = 144)	4	Browser	No	Closed
*Tragulus nigricans*	Artiodactyla	Ruminantia	?	12 (n = 50)	4	Browser	No	Closed
*Catagonus wagneri*	Artiodactyla	Suoidea	?	46 (n = 47)	35	Browser	No	Closed
*Phacochoerus africanus*	Artiodactyla	Suoidea	55 (n = 12)	46 (n = 75)	73	Grazer	Yes	Open
*Potamochoerus porcus*	Artiodactyla	Suoidea	45 (n = 1)	53 (n = 30)	85	Other	Yes	Closed
*Sus barbatus*	Artiodactyla	Suoidea	?	55 (n = 7)	97	Other	?	Closed
*Sus cebifrons*	Artiodactyla	Suoidea	?	51 (n = 14)	28	Browser	?	Closed
*Sus scrofa*	Artiodactyla	Suoidea	37	47 (n = 79)	200	Other	No	Closed
*Tayassu pecari*	Artiodactyla	Suoidea	?	43 (n = 21)	33	Other	No	Closed
*Camelus bactrianus*	Artiodactyla	Tylopoda	15 (n = 2)	2 (n = 136)	570	Mixed	No	Open
*Camelus dromedarius*	Artiodactyla	Tylopoda	13 (n = 3)	-1 (n = 127)	450	Browser	No	Open
*Lama guanicoe*	Artiodactyla	Tylopoda	?	15 (n = 89)	120	Grazer	No	Open
*Lama lama*	Artiodactyla	Tylopoda	?	9 (n = 48)	94	Grazer	No	Rocky
*Vicugna pacos*	Artiodactyla	Tylopoda	23 (n = 1)	17 (n = 55)	66	Grazer	No	Open
*Vicugna vicugna*	Artiodactyla	Tylopoda	17 (n = 1)	20 (n = 66)	50	Grazer	No	Rocky
*Equus asinus*	Perissodactyla	Equidae	53 (n = 2)	58 (n = 88)	275	Mixed	No	Open
*Equus burchelli*	Perissodactyla	Equidae	47 (n = 2)	59 (n = 109)	280	Grazer	No	Open
*Equus caballus*	Perissodactyla	Equidae	44 (n = 4)	59 (n = 135)	250	Grazer	No	Open
*Equus grevyi*	Perissodactyla	Equidae	?	66 (n = 182)	401	Grazer	No	Open
*Equus hemionus*	Perissodactyla	Equidae	?	58 (n = 117)	325	Grazer	No	Open
*Equus zebra*	Perissodactyla	Equidae	50 (n = 1)	61 (n = 156)	274	Grazer	No	Open
*Ceratotherium simum*	Perissodactyla	Rhinocerotidae	38 (n = 9)	57 (n = 140)	1930	Grazer	No	Open
*Dicerorhinus sumatrensis*	Perissodactyla	Rhinocerotidae	25 (n = 1)	?	1400	Browser	?	Closed
*Diceros bicornis*	Perissodactyla	Rhinocerotidae	36 (n = 4)	35 (n = 80)	1129	Browser	No	Open
*Rhinoceros sondaicus*	Perissodactyla	Rhinocerotidae	28 (n = 2)	?	1890	Browser	?	Semi-aquatic
*Rhinoceros unicornis*	Perissodactyla	Rhinocerotidae	34 (n = 2)	17 (n = 83)	1416	Mixed	No	Open
*Tapirus bairdii*	Perissodactyla	Tapiridae	?	21 (n = 34)	200	Browser	No	Semi-aquatic
*Tapirus indicus*	Perissodactyla	Tapiridae	14 (n = 1)	28 (n = 117)	263	Browser	No	Semi-aquatic
*Tapirus terrestris*	Perissodactyla	Tapiridae	25 (n = 3)	23 (n = 149)	200	Browser	No	Semi-aquatic

Data on body mass, diet, head-butting behaviour, and habitat are from the literature (see Supplementary Table S1). n represents the number of measurements.

The bony labyrinth is one of the first organs to completely ossify in mammals as its adult size and shape are reached at mid-gestation^50,51^. However, the orientation of the LSC seems to show age-related variations in some tetrapod species, including humans, which may impact their head posture^26,52,53^. As such, juveniles were excluded from the dataset.

### Head posture from dry skulls

A total of 285 medical quality CT-scans and micro-CT scan representing 118 species mostly from the American Museum of Natural History (AMNH), Ditsong Museum (AZ and TM), Evolutionary Studies Institute of the University of the Witwatersrand (BP), Wits Life Science Museum (WLSM), School of Anatomical Science of the University of the Witwatersrand (MS and ZA), Natural History Museum of Basel (NMB), Yale Peabody Museum of Natural History (YPM), and Zoological Museum of the University of Zurich (ZM) (see the Supplementary Table S1 for details), were used.

The bony labyrinths of each skull were segmented manually and reconstructed in 3D using the software AVIZO 9 (FEI VSG, Hillsboro OR, USA) at the virtual imaging labs of the Evolutionary Studies Institute and the Natural History Museum of Basel. The skull was reconstructed using either the Isosurface or threshold functions under the same software. The angle between the plane of the LSC and the main axis of the skull was then measured in lateral view in 2D (Fig. 2b). The plane of the LSC was determined visually in lateral view, following most previous authors^19,20,22,24–26^. The main axis of the skull was traced as the axis running from just above the premaxilla (approximately at the level of the centre of the nasal opening) to the middle of the occiput (Fig. 2b) in order to maximize the homology with the measurements taken on living animals. This angle represents the anterior tilting of the head if the LSC is considered horizontal. This angle is hereafter referred to as “the reconstructed cranial orientation” or “reconstructed head posture”. Measurements were taken bilaterally when both bony labyrinths were available and then averaged for each species (Table 1). For consistency, all measurements were taken by the same author (J.B.). None of the samples expressed strong lateral tilting of the LSC or an undulating morphology that could impede taking this measurement or affect its accuracy. The intraspecific standard deviation (measurement error) for reconstructed head posture is ± 2.1°.

The complete dataset of reconstructed head postures is available in the Supplementary Table S1. This dataset was complemented by measurements made on the published pictures from Girard and Schellhorn^5,30^ (see Supplementary Table S1).

As for the picture dataset, only the individuals showing reasonable signs of maturity (e.g. cranial bone fusion, erupted molars) were considered.

### Data processing

The dataset was analyzed using phylogenetic comparative methods to control for the non-independence of observations^54–56^. We used the time-calibrated phylogenetic tree of mammals of Bininda-Emonds et al.^57^ because it encompasses all the species in our dataset and fossils can be easily added to it in future analyses. The tree was pruned to match the species in our dataset using function ‘drop.tip’ in R package ape^58^. All subsequent analyses were performed in R v3.6.3 (R Core Team, 2020). The phylogenetic signal of individual variables was estimated using Pagel’s lambda^59^ for continuous features (reconstructed cranial orientation, neutral head posture, body mass) using function ‘phylosig’ in package phytools^60^. Lambda was chosen over the other commonly used estimator K^61^ because of the latter’s poor performance for trees with small sample sizes and polytomies^62,63^, both of which can be found in our dataset. For binary traits (head-butting; see below), phylogenetic signal was estimated with the D-statistic^64^ using function ‘phylo.d’ in package caper^65^. To test whether the plane of the LSC can be used as a reliable proxy to reconstruct the neutral head posture, we regressed the neutral head posture of living animals on the reconstructed cranial orientation using data in Table 1, and phylogenetic generalized least squares (PGLS) regressions^66,67^. PGLS were compiled using the ‘gls’ function in package nlme^68^, with correlation structures for each evolutionary model specified in ape^58^. A model selection procedure based on the corrected Akaike information criterion (AICc) was applied to the regressions using the package AICcmodavg^69^. Five evolutionary models were considered for this selection procedure (see^70^): Brownian Motion, Pagel’s Lambda, Ornstein–Uhlenbeck, Early Burst, and White Noise – i.e. non-phylogenetic, ordinary least squares (OLS) regression. All regressions were performed using raw and log-transformed data (natural logarithm). Both variables in the models are in the same unit and order of magnitude, and the models built with raw data showed a higher significance and met parametric assumptions better than models built with log-transformed data. For this reason, we used the former to assess the relationship between the two variables.

Because of the high degree of body mass allometry in neuroanatomical features^71–73^, body mass measurements for all species in the sample were taken from the literature (Supplementary Table S1) and included as a co-predictor to be tested against models built with only the reconstructed and neutral head postures as predictors in the AICc-based model selection procedures. The coefficient of determination and p-value for generalized least squares regressions cannot be compiled straightforwardly due to the autocorrelated structure of the residuals^67^. Following Paradis^55^, we compiled a pseudo-R-squared and p-value based on McFadden’s formula^74^, based on a likelihood ratio test between our model and a null model. Normality and homoscedasticity of the residuals were assessed using a Shapiro–Wilk test and a Q-Q plot, and graphically using residuals v. fit plots, respectively^75^.

Finally, phylogenetic one-way Analyses of Variance (phylANOVA)^76^ with False Discovery Rate posthoc corrections^77^ were used to test for a difference between groups in three separate factors, for both reconstructed cranial orientation and neutral head posture (Table 1). The first factor is diet, for which species were categorized as “browser”, “grazer”, “mixed” (for a mixed diet between browsing and grazing), or “other” (for omnivorous and myrmecophagous species). The second predictor is whether a species practices head-to-head combat. The head-butting category includes wrestling and ramming species (hereafter referred to simply as head-butting species) but excludes flank-butting species (e.g. giraffes). The last predictor is the habitat, which was scored between open (savannah or steppes), closed (forest or jungle), mixed (mix of open and closed habitats), rocky (for species living on steep, rocky slopes), or semi-aquatic. The scoring of all three predictors was done using the literature (see the list in Table 1). PhylANOVAs were performed using function ‘phylANOVA’ in phytools^60^.

### Ethics declarations

As the animals were not approached or armed, no ethical clearance was necessary for this study.

## Results

All the data in the dataset for which a phylogenetic signal could be measured (neutral head posture, reconstructed cranial orientation, body mass, and head-butting) carry a strong phylogenetic signal (lambda > 0.8 for the first three variables; D = − 0.2841056 for head-butting) (See Supplementary Table S1).

**Figure 3:**
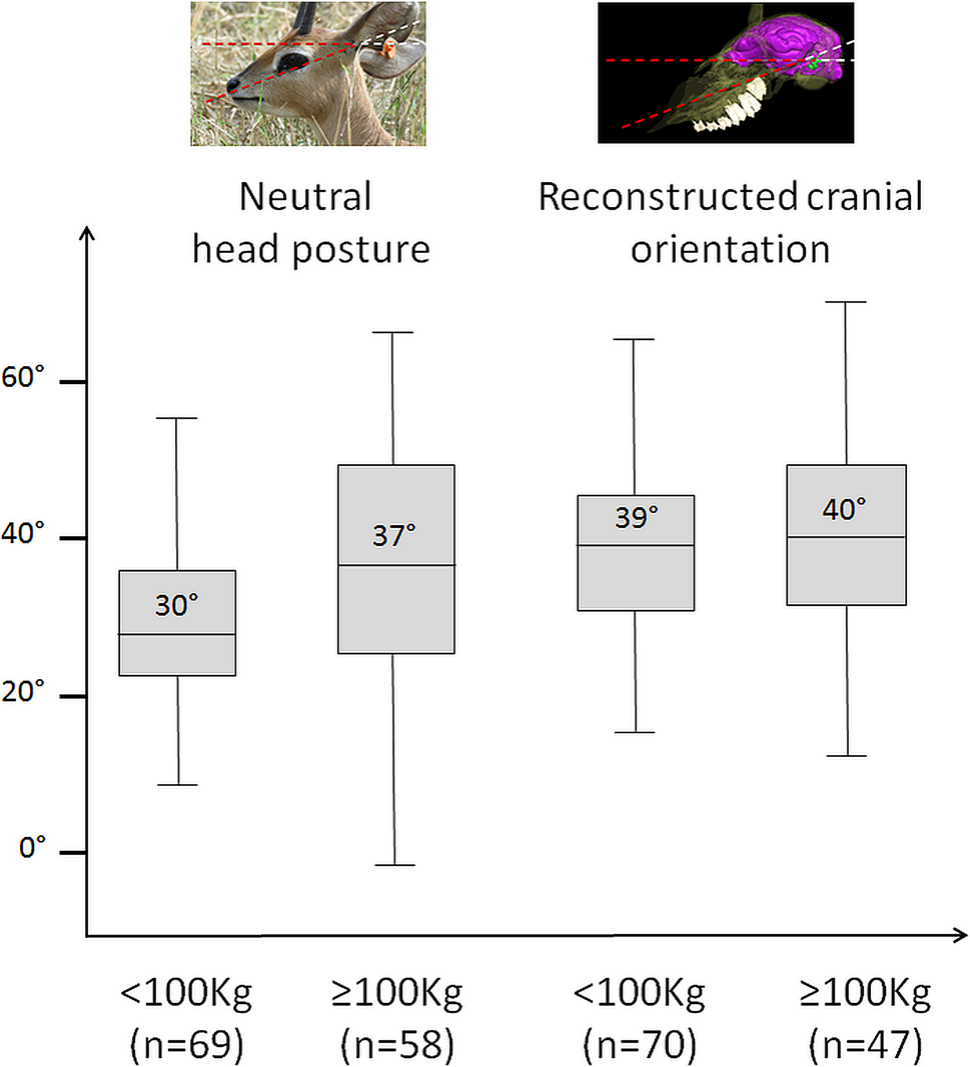
Boxplot of neutral head posture (left) and reconstructed cranial orientation (right) in degrees (°) in species with body mass below 100 kg (< 100 kg) and species with body mass superior or equal to 100 kg (≥ 100 kg). The average angle for each category is indicated in the corresponding boxplot. n represents the number of species in each category.

Species with body mass under 100 kg have an average neutral head posture of 30° and reconstructed cranial orientation of 39°, whereas species larger than 100 kg have a neutral head posture averaging 37° and average cranial orientation of 40°. This suggests an effect of body mass on head posture as was hypothesized by Köhler^47^, but not on the orientation of the LSC (Fig. 3). This is consistent with statistical analyses, which identify a very weak effect of body mass on neutral head posture (R^2^ = 0.040; p-value = 0.014), and none on reconstructed cranial orientation (R^2^ = 0.023; p-value = 0.054) using OLS. However, once corrected for phylogeny using PGLS, the effect of body mass on head posture (R^2^ = 0.030, p-value = 0.06025) and cranial orientation (R^2^ = 0.012, p-value = 0.9903) is no longer significant.

**Figure 4:**
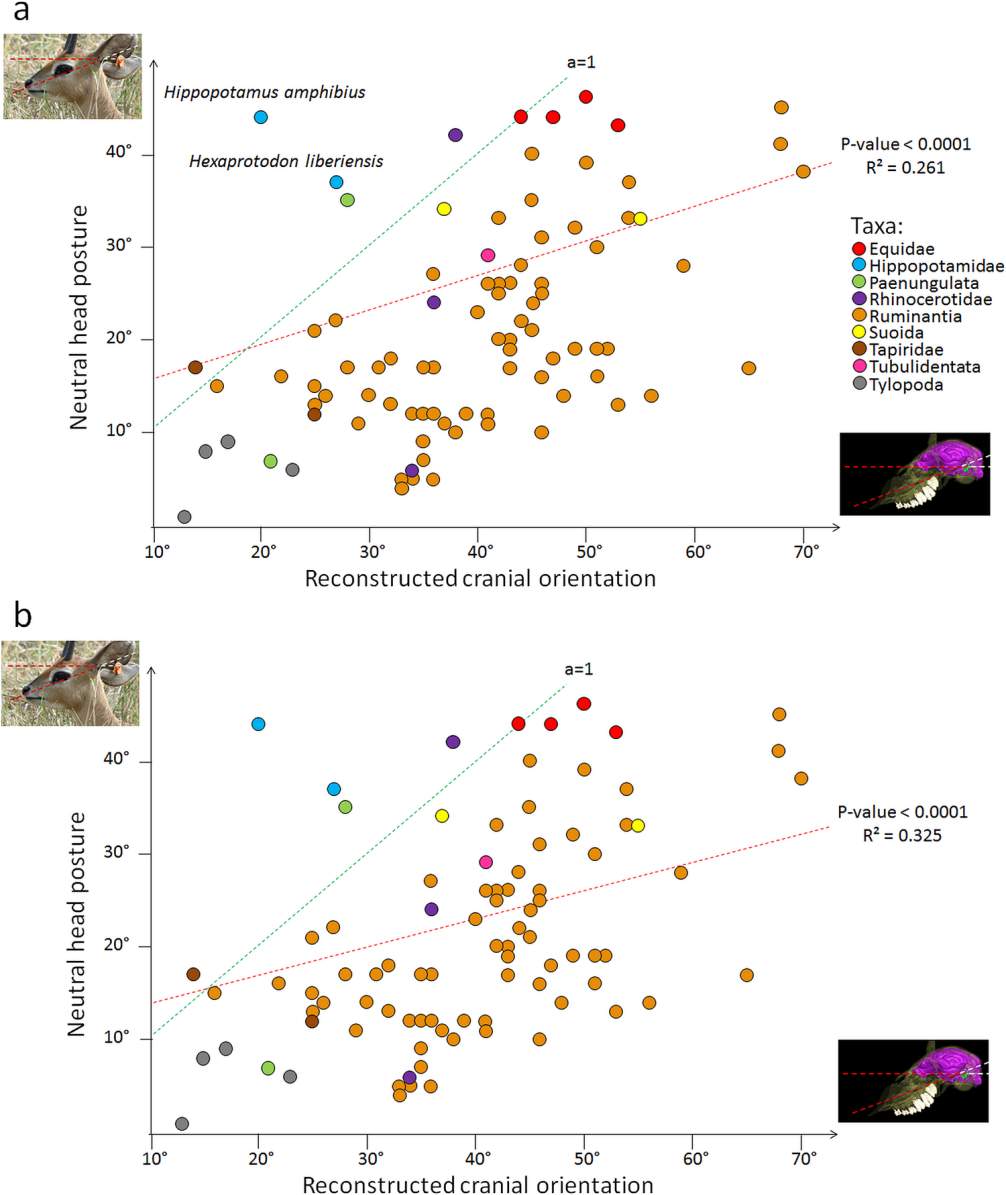
Phylogenetic regressions of neutral head posture of living mammals plotted against the reconstructed cranial orientation (in °) without body mass (**a**) and with body mass included as a co-predictor (**b**). Abbreviations: R^2^, coefficient of determination.

Phylogenetic regressions (Fig. 4a) identify a statistically significant (p-value = 4.519e−07) but relatively low correlation (R^2^ = 0.261) between neutral head posture and the reconstructed cranial orientation. This supports that the orientation of the LSC in life is correlated to the neutral head posture in “ungulates”. The equation of the linear model is:

1 $$ {\text{ Neutral head posture }}\left(^\circ \right)\, = \,0.{384}\, \times \,{\text{reconstructed cranial orientation }}\left(^\circ \right)\, + \,{13}.{468}. $$

The 95% confidence interval for the slope (0.242–0.526) is significantly different from 1, which means that this model cannot be approximated to an isometric relationship (which would be expected if the LSC was held horizontally).

The model including body mass, neutral, and reconstructed head postures, and the interaction term of the three as co-predictors was selected by AICc as fitting our data best (Fig. 4b). This model shows results very similar to those of the simple regression model, being significant with a slightly stronger correlation (R^2^ = 0.325; p-value = 3.235e−07). The equation of the resulting model is written:

2 $$ {\text{ Neutral head posture }}\left(^\circ \right)\, = \,{11}.{677}\, + \,0.{328}\, \times \,{\text{reconstructed cranial orientation }}\left(^\circ \right) - 0.00{1}\, \times \,{\text{body mass }}\left( {{\text{kg}}} \right) - 0.000{2}\, \times \,{\text{reconstructed cranial orientation }}\left(^\circ \right) \, \times {\text{ body mass }}({\text{kg}}). $$

The slope confidence interval is slightly lower than that of the previous model (for reconstructed cranial orientation: 0.148–0.508), which removes it even further from an isometric relationship (Fig. 4b). The very low coefficients for body mass and reconstructed orientation × body mass are not significantly different from zero (see Supplementary Table S1), and are reported here to ensure full transparency of our results.

Surprisingly, for both simple and multiple regression models, the evolutionary model selected by AICc was the Early Burst (EB) model^61^, representing a rapid adaptive radiation followed by stasis. EB models are known to be rarely selected as the best evolutionary model in such selection procedures^78^.

**Figure 5:**
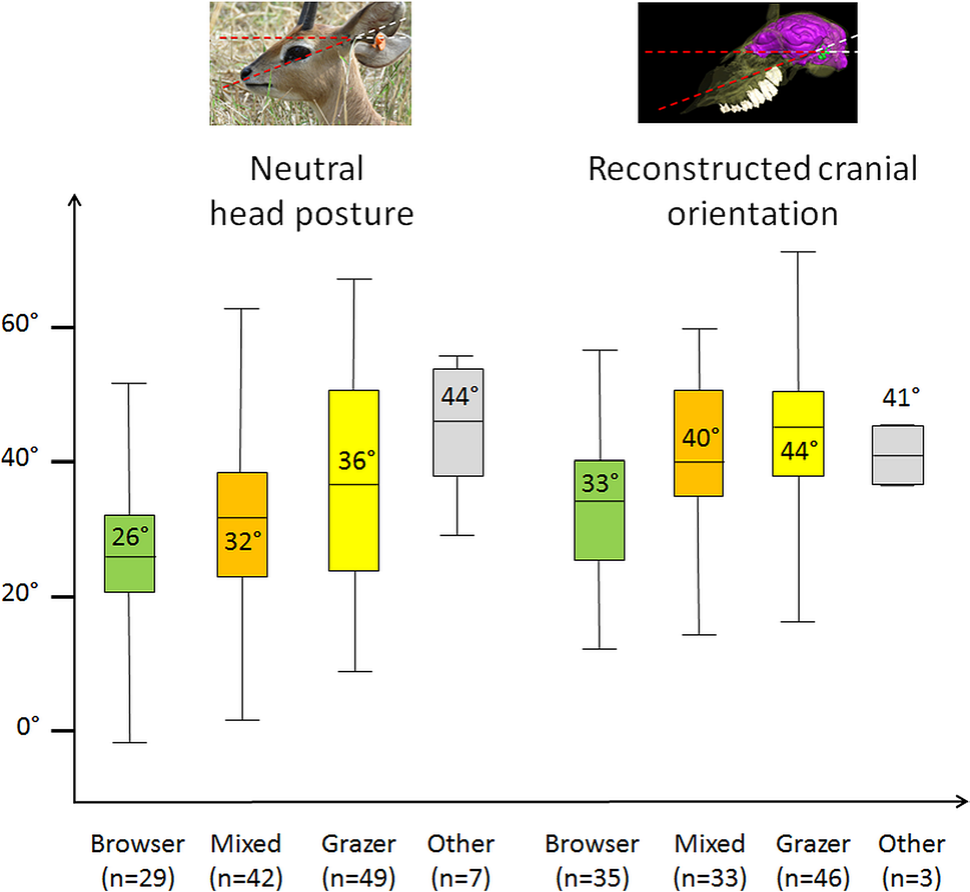
Boxplot of neutral head posture (left) and reconstructed cranial orientation (right) in degrees (°) in browsers (green), mixed feeders (orange), grazers (yellow), and omnivorous + myrmecophagous species (other) (grey). The average angle for each category is indicated in the corresponding boxplot. n represents the number of species in each category.

On average, browsers tend to hold their heads less tilted anteriorly (26°) than mixed feeders (32°), and grazers (36°) in neutral posture (Fig. 5). This seems to reflect on the reconstructed cranial orientation as browsers have a higher reconstructed head posture (33°), than mixed feeders (40°) and grazers (44°) (Fig. 5). Phylogenetic ANOVAs indicate a significant difference between browsers and grazers for the reconstructed orientation of the skull (F = 7.723; p-value = 0.046), but not for the neutral head posture (F = 2.663; p-value = 0.516). Mixed feeders are statistically indiscernible from both browsers and grazers in any case.

**Figure 6:**
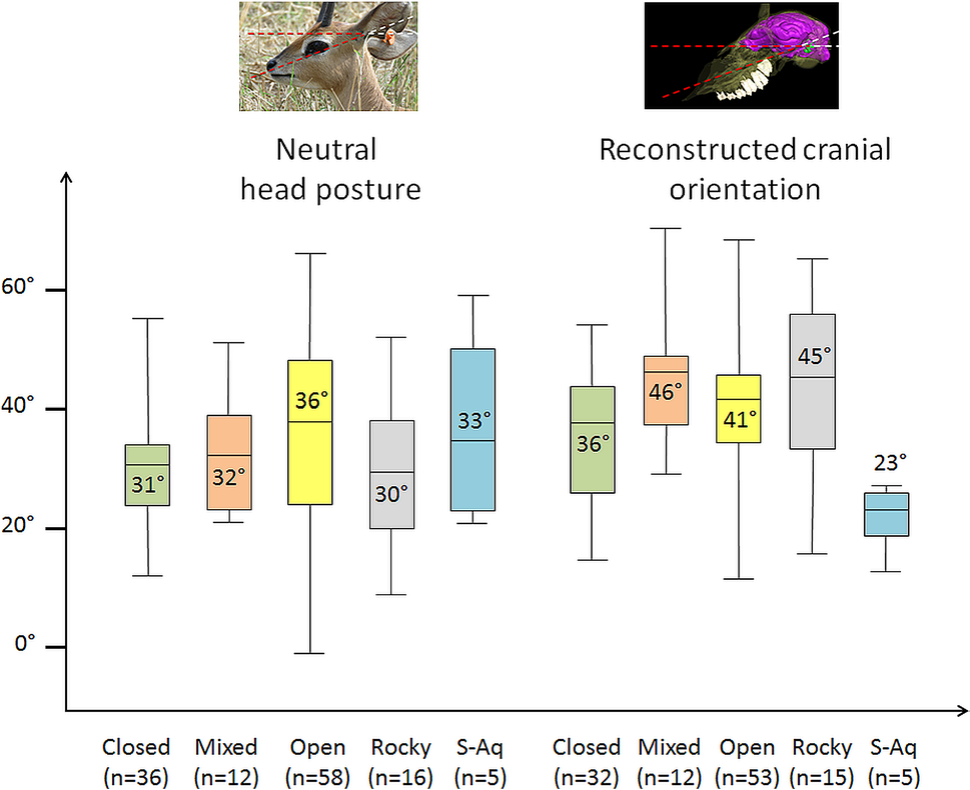
Boxplot of neutral head posture (left) and reconstructed cranial orientation (right) in degrees (°) in species living in closed (green), mixed (orange), open (yellow), rocky (grey), and semiaquatic (S-Aq) (blue) habitats. The average angle for each category is indicated in the corresponding boxplot. n represents the number of species in each category.

Variations of reconstructed cranial orientation do not show any trend with habitat preference (Fig. 6), but a slight trend toward more downwardly tilted head postures seems to occur in species living in a more open habitat (Fig. 6); however, this trend is not significant (F = 1.343; p-value = 0.792). Semi-aquatic species seem to have a more posteriorly tilted LSC resulting in a higher reconstructed cranial orientation (23°) than fully terrestrial species, but this does not reflect on the neutral head posture (Fig. 6). Unfortunately, the sample size for this category was too low to effectively test if this difference was significant or simply the result of the scarcity of semi-aquatic species in the dataset.

**Figure 7:**
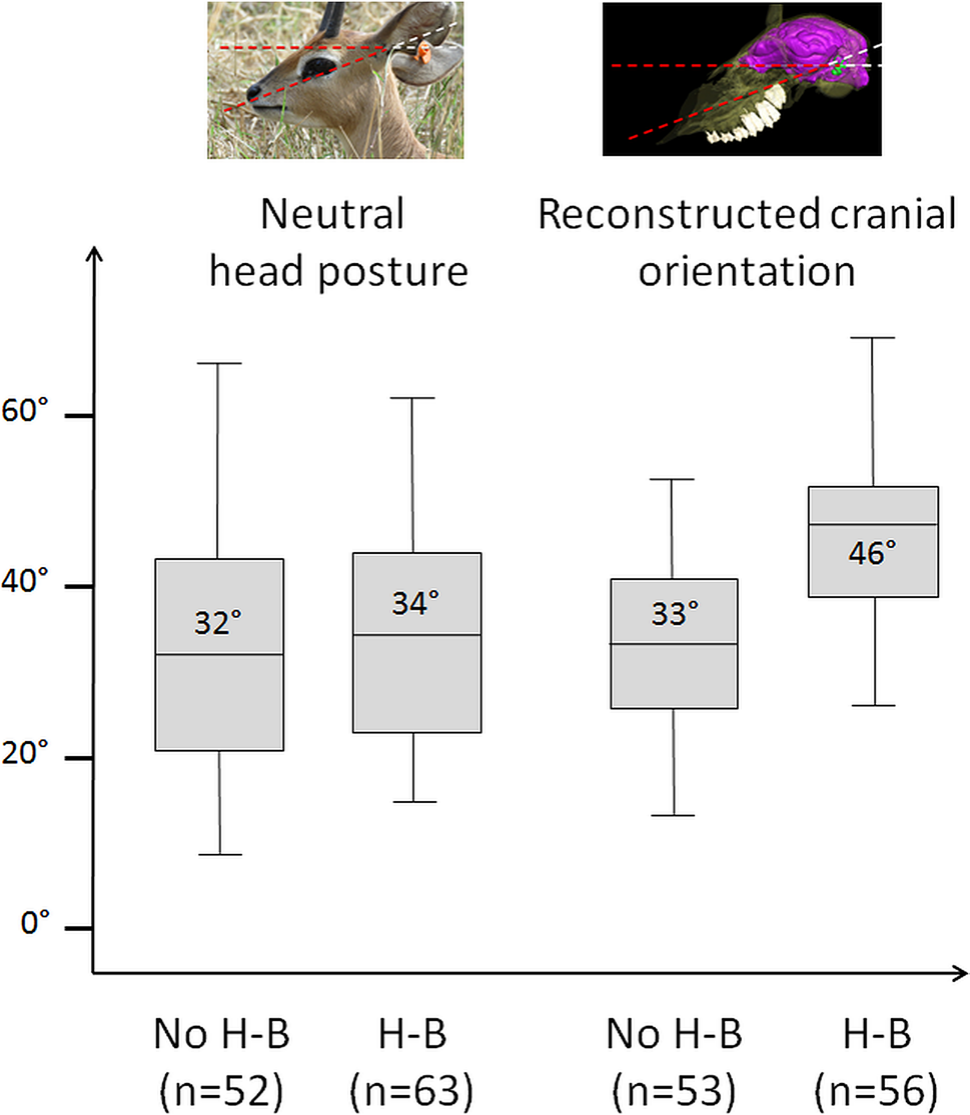
Boxplot of neutral head posture (left) and reconstructed cranial orientation (right) in degrees (°) in non-head-butting (No H-B) and head butting (H-B) species. The average angle for each category is indicated in the corresponding boxplot. n represents the number of species in each category.

Head-butting is found to have a highly significant statistical effect on reconstructed head posture (F = 39.467; p-value = 0.002), with a difference of 13° between head-butting and non-head-butting species on average (Fig. 7); however, with only 1° difference on average (Fig. 7), the same is not true for the neutral head posture (F = 3.126; p-value = 0.591).

## Discussion

### LSC orientation is correlated with head posture, but is not horizontal

The assumption that the plane of the LSC can be used as a reliable indicator of the horizontal plane on dry skulls and thus can serve as a proxy to reconstruct the “habitual” or “alert” head posture in extinct species has been a long-held^17^, yet insufficiently tested hypothesis in mammals. Attempts to test this hypothesis have highlighted that the plane of the LSC is often tilted upward compared to the horizontal in most mammalian species^5,6,11,30,45^. A famously baffling example is that of humans, in which aligning the plane of the LSC to the horizontal plane results in a “habitual” posture of the head inclined 30° down anteriorly^1–6,8,11,12,18,46^. In the current study, the steenbuck (*Raphicerus campestris*) is the only species in which the average neutral and reconstructed head postures are the same (Table 1), which means that, on average, the LSC is parallel to the horizontal plane when the steenbuck’s head posture is neutral. Among the species for which the neutral head posture and reconstructed cranial orientation could be compared, only half of them show a difference between the averages of the two that is below 10°. As such, even though the plane of the LSC should be horizontal on theoretical grounds^13^, this is not the rule in “ungulates”.

The two phylogenetic regressions provided here (Fig. 4) are the first large sample size attempts to address the existence and nature of a correlation between the orientation of the plane of the LSC and the neutral head posture in mammals across a large taxonomic sampling. We find that whether corrected or not for body mass, the correlation between the reconstructed cranial orientation and the neutral head posture is significant (p-value < 0.0001); however, if the plane of the LSC was held horizontally in neutral head posture, the regression line should not differ significantly from an isometric line. Instead, both regression lines have slopes that significantly differ from 1 (Fig. 4), which means that they cannot be approximated by isometric lines. For this reason, though there is a significant correlation between the orientation of the LSC and that of the head in “ungulates”, the plane of the LSC should not be considered horizontal when reconstructing ancient head posture.

According to the phylogenetic regressions, the equation that describes the relationship between the reconstructed cranial orientation and the neutral head posture is given in Eq. (1), and that between cranial orientation, head posture, and body mass is given in Eq. (2). As estimating body mass in extinct species is always contentious^79–82^, the first of these equations may seem more practical to estimate the actual head posture of a given extinct ungulate species. In both cases, the variance of the residuals is high, which might indicate a low predictive power of these models (R^2^ equals 0.26 and 0.33, respectively).

The misalignment between the plane of the LSC and the horizontal is consistent with the results obtained by Marugán-Lobón et al.^43^ on birds using Duijm’s dataset^8^, though their approach to the study of head posture was different from the one presented here, which limits comparisons. The reason why the LSC would not be aligned with the horizontal in the neutral posture is still unclear. It may be explained by the very function of the canals and ampullae, which are meant to record head movements and play crucial roles in the vestibulo-ocular and vestibulo-collic reflexes to compensate for the movements and accelerations of the head compared to the eyes and the rest of the body^6,13,14,43^. As such, recording movements and monitoring reflexes during locomotion and head movements along the predominant axis of yaw would be the main drivers of LSC adaptations^83^, which would result in a relaxed selection on the functions performed when the head remains still, such as aligning the plane of the LSC to the horizontal plane during neutral head posture. In support of this hypothesis, a recent study by Dunbar et al.^84^ on the orientation of the LSC during locomotion in horses found a 66° inclination of the head below the horizontal during slow walk, and a higher head posture of 56° and 55° during trot and canter. They argued that fast locomotion brought the plane of the LSC to about 5° around the horizontal in their specimens^84^. According to Zubair et al.^85^, domestic cats keep their LSC about 10° to the horizontal during locomotion, although Hullar^12^ reports a tilting up to 60° of the LSC in cats during “normal activities”. Primates appear to keep the plane of their LSC within a 20° range about the horizontal during locomotion^84^, which is consistent with its orientation at rest^12^. Published data about the orientation of the LSC during locomotion are scarce and some are contradictory. Additionally, they are difficult to acquire as animals rarely maintain a static head posture during locomotion as they often pitch their heads repeatedly^84,85^. In the future, such data could nevertheless enable testing whether the orientation of the LSC would be a better predictor of the head posture during locomotion rather than at rest.

Another hypothesis is that an overall misalignment of all three semicircular canals would enable all semicircular canals to record a component of horizontal and vertical accelerations^43^.

### The effect of phylogeny

Both the neutral and reconstructed head postures carry an important phylogenetic signal (Lambda equals 0.97 and 0.84, respectively). For paleontologists, this strong phylogenetic signal implies that the best way to predict the head posture of an extinct “ungulate” is to look at the neutral head posture of its modern relatives. In comparison, once the data are corrected for phylogeny, diet is found to have only a weak correlation with the reconstructed cranial orientation (F = 7.723; p-value = 0.046), and no significant effect on the neutral head posture (F = 2.663; p-value = 0.516). This reflects well in the dataset.

**Figure 8:**
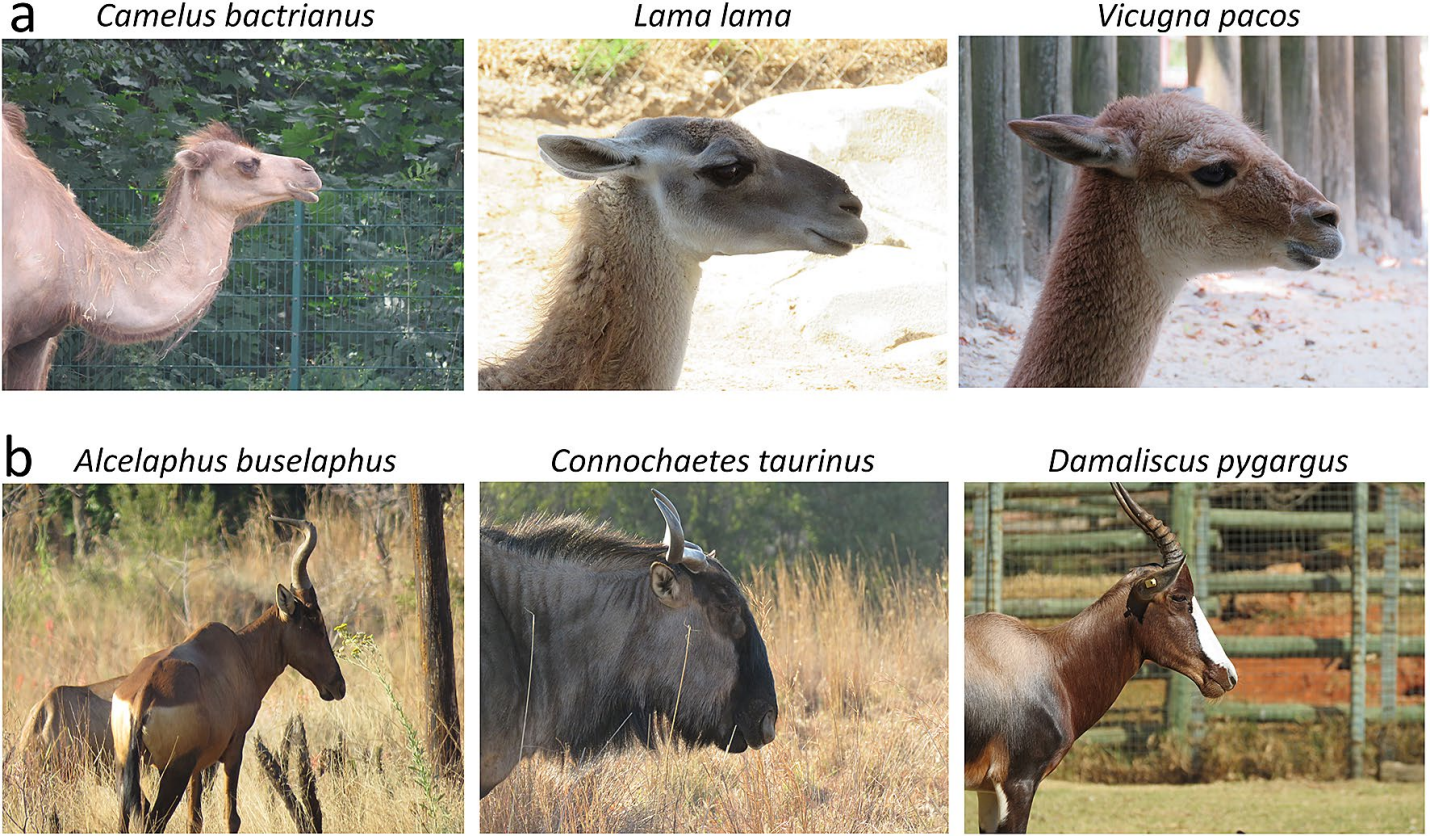
Neutral head posture in three species of Tylopoda (**a**) and Alcelaphinae (**b**) contrasting the almost horizontal head posture assumed by *Camelus*, *Lama,* and *Vicugna* with the almost vertical head posture of *Alcelaphus*, *Connochaetes,* and *Damaliscus*.

The Tylopoda is the group in which the head is the most consistently tilted upward, with an average neutral head posture of 10°. This reflects on their reconstructed cranial orientation which averages 17°. Tylopods nevertheless include grazers (genus *Vicugna*), mixed feeders (*Camelus bactrianus*), and browsers (*Camelus dromedarius*) that all keep their heads relatively high (Figs. 4, 8a). On the other end of the spectrum, pigs display remarkable consistency at holding their head low (average neutral head posture for Suoidea = 46°), with for example the grazing *Phacochoerus* and the browsing *Catagonus* both keeping their head 46° below the horizontal plane on average (Table 1, Fig. 4).

The species that holds their head the lowest below the horizontal belong to the Equidae (average neutral head posture = 60°; average reconstructed cranial orientation = 49°) and Alcelaphinae (average neutral head posture = 57°; average reconstructed cranial orientation = 61°) (Figs. 4, 8b). The species with the most tilted head posture is the Grevy’s zebra (*Equus grevyi*), with a 66° tilt on average (Table 1), comparable to the extreme 67° reconstructed cranial orientation of the sauropod dinosaur *Nigersaurus*^20^. Tilting of the head was hypothesized to be correlated to body size in “ungulates”^47^, with small, forest-dwelling species holding their head higher than large species adapted to savannah. A similar trend is found here, with species above 100 kg having their head tilted 37° anteriorly on average whereas species below 100 kg hold their heads 30° below the horizontal on average (Fig. 3). Statistical tests on our dataset find a significant correlation between body mass and head posture (p-value = 0.014), but this correlation is no longer significant once the data are corrected for the effect of phylogeny (p-value = 0.060). This strongly suggests that the trend observed here and by Köhler^47^ actually reflects the fact that large savannah herbivores belong to just a few clades (e.g. equids, alcelaphins, and hippotragins) whereas the small ones mostly belong to the Antilopinae.

A more significant effect of body mass might nevertheless be found while including very small-bodied species (e.g. rodents, shrews) because their head posture would be more constrained by its proximity to the substrate. Similarly, species with a sprawling posture would also have to keep their head higher, as already observed in many reptiles^12,41–43^.

The relationship between LSC orientation and phylogeny has been empirically anticipated as the way the LSC enters the vestibule (either directly above the posterior ampulla or at different levels within the ampulla) is distinctive between different clades of ruminant^50,51,86–88^.

The selection of an Early Burst model as the best fit for the whole dataset suggests that the evolution of head posture might represent a classic example of adaptive radiation^61,78^. This may be the effect of the abundance of bovids in our dataset, which originated in the early Neogene and rapidly adapted to a wide variety of ecological niches, diet, body mass, social behavior, and habitat^48,89^. However, the presence of a strong phylogenetic signal for both head posture variables cannot be directly interpreted as evidence for such a radiation in terms of evolutionary process, which would require further analyses of diversification rates^90^. The increase of phenotypic divergence resulting in different niches for a given character in a clade does not always correspond to an adaptive radiation, even when it closely matches the underlying phylogeny. This is due to ecological interactions between distantly related species, which can result in a similar timing of evolutionary shifts for distinct clades, the detail of which is often very difficult to decipher without exhaustively sampling each of these clades^91^. Indeed, even if an early radiation in head posture diversity would be consistent with the high discrepancy between taxonomic groups for both variables (Fig. 4), the small sample size for all groups except Ruminantia prevents a straightforward discussion of specific evolutionary constraints in that context. Furthermore, other factors such as a high proportion of sympatric species in the sample may also artificially increase the fit of an EB evolutionary model^78^. A larger and broader sampling among mammals is thus likely to blur such a signal and could result in a more homogeneous distribution of residuals that would not necessarily match so closely the observed pattern of relative phylogenetic proximity.

### Diet

As grazers have to keep their head low while foraging on grass, whereas browsers have to catch leaves higher in bushes and trees, and because herbivores spend most of their time acquiring low-energetic food^49,92,93^, it is expected on an evolutionary scale that the skull, neck musculature, and vestibular apparatus of herbivores would adapt to these different feeding strategies and that it would reflect in their head posture, even at rest^20,30,45^. As such, a gradually more anteriorly tilted neutral head posture is expected as moving from browsing species to mixed feeders, and finally grazers^20,30^. Schellhorn^30^ was the first to compare the reconstructed cranial orientation of Rhinocerotidae to their actual head posture (using an open-access database of photographs) and found results consistent with such a gradient.

**Figure 9:**
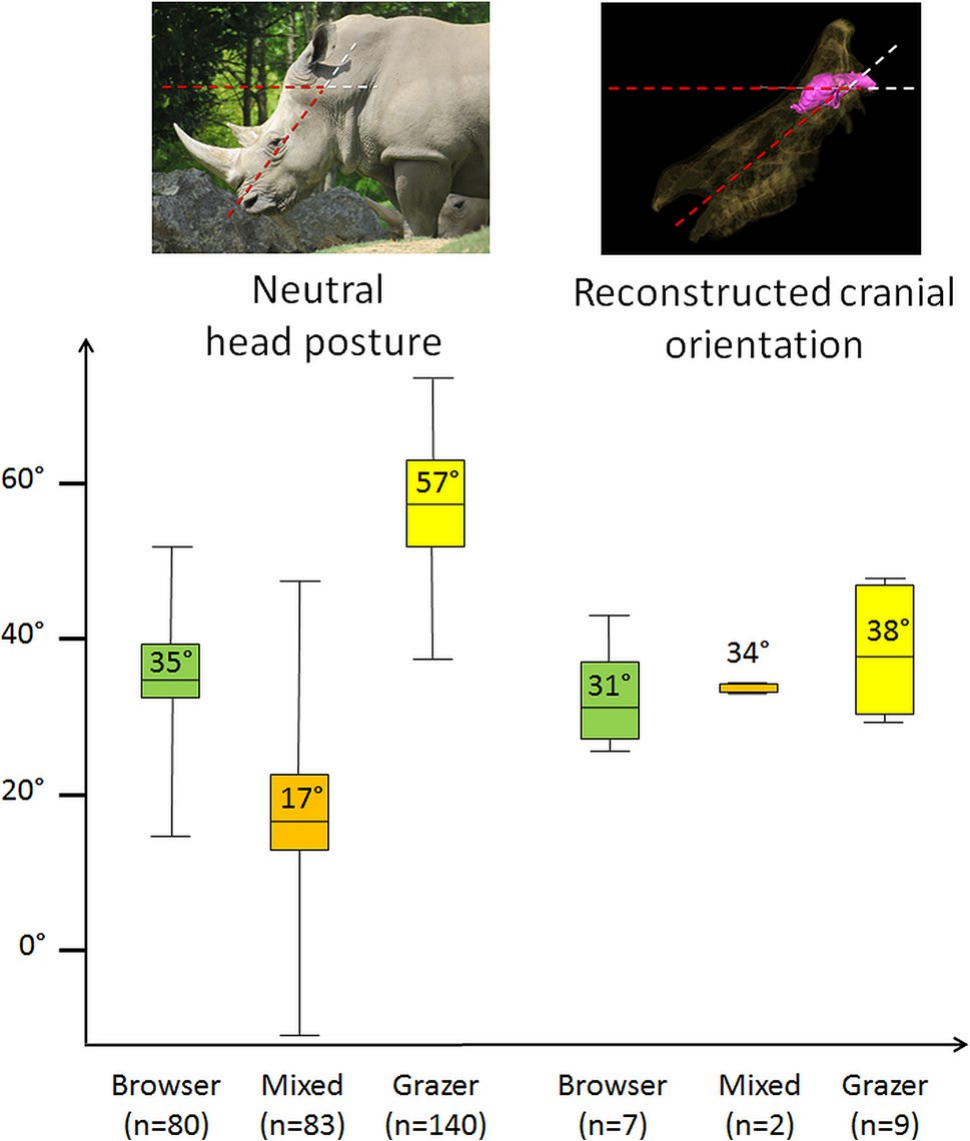
Boxplot of neutral head posture (left) and reconstructed cranial orientation (right) in degree (°) in browsing (green), mixed feeding (orange), and grazing (yellow) Rhinocerotidae. The average angle for each category is indicated in the corresponding boxplot. n represents the number of measurements in each category.

**Figure 10:**
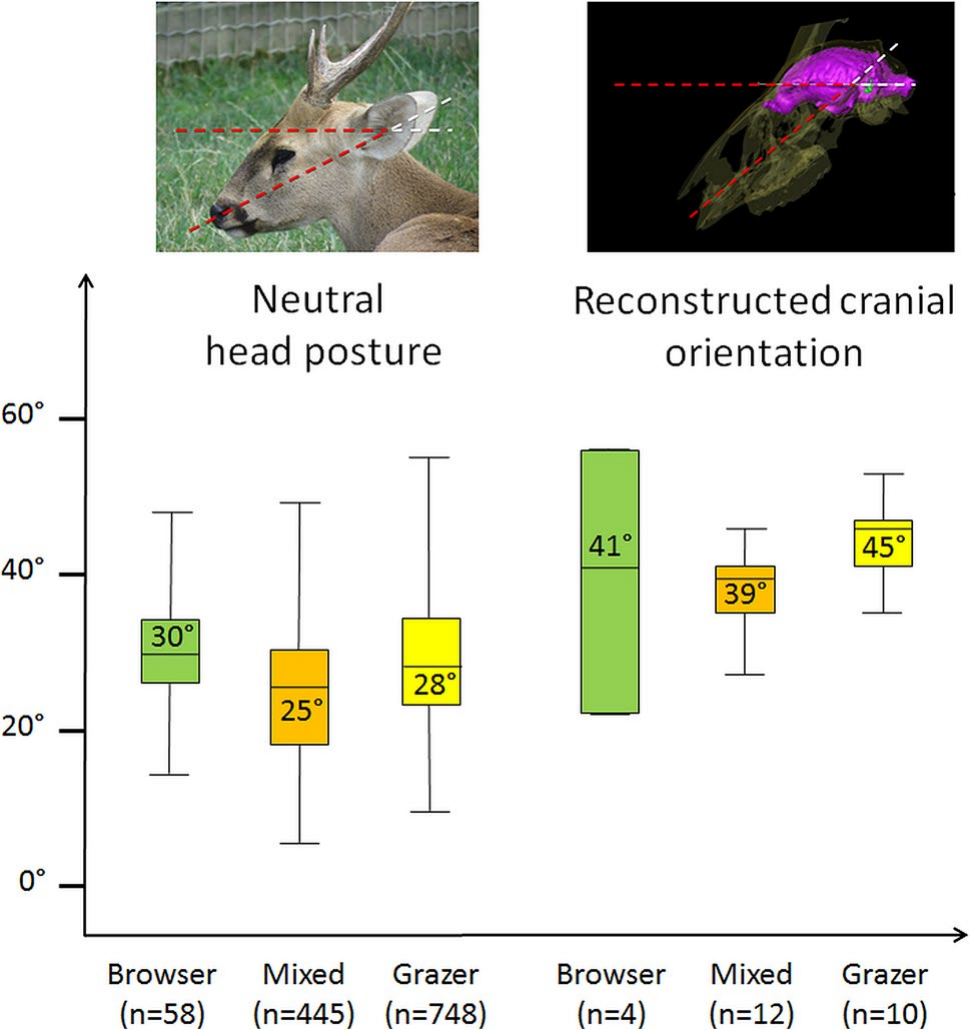
Boxplot of neutral head posture (left) and reconstructed cranial orientation (right) in degrees (°) in browsing (green), mixed feeding (orange), and grazing (yellow) Cervidae. The average angle for each category is indicated in the corresponding boxplot. n represents the number of measurements in each category.

Measuring the reconstructed cranial orientation from Schellhorn’s published figures and adding our own observations of neutral head posture, we do find a more tilted reconstructed cranial orientation in the grazing *Cerathotherium* (average reconstructed cranial orientation = 38°), than in the mixed feeder *Rhinoceros* (average reconstructed cranial orientation = 34°), and the browsing *Dicerorhinus* and *Diceros* (average reconstructed cranial orientation = 31°) (Fig. 9); however, this gradient does not reflect on the neutral head posture that shows no particular trend among rhinocerotids (Fig. 9). Despite the large difference between the average head posture of browsers and grazers, the very low head posture of mixed feeder rhinocerotids casts some doubts on the validity of the correlation between head posture and diet (Fig. 9). The Cervidae constitutes a more striking example (Fig. 10). In cervids, the head is consistently kept within about 10° around the average neutral head posture (20°) regardless of diet, and browser and grazers are not distinguishable based on neutral head posture (average = 30° for browser; average = 28° for grazers) or reconstructed cranial orientation (average = 41° for browsers; average = 45° for grazers) (Table 1).

The situation in rhinocerotids illustrates that for the whole dataset. Diet is found to have no statistical effect on neutral head posture (p-value = 0.516), and its relationship to the reconstructed cranial orientation is barely significant (p-value = 0.046). Average values for neutral and reconstructed head postures show a visible increase in tilting with a more grass-rich diet, but there is a strong overlap between dietary groups for both variables (Fig. 5). This suggests that even though diet could potentially be reconstructed in extinct species using the orientation of the LSC, caution should be taken as i) browsers and grazers could be statistically discriminated, but mixed feeders could not; ii) the correlation between reconstructed cranial orientation and diet does not seem to reflect on the neutral head posture. The reason why remains unknown; and iii) the high p-value suggests that adding more data (particularly CT data) in the future may affect this correlation.

### Semi-aquatic adaptation

**Figure 11:**
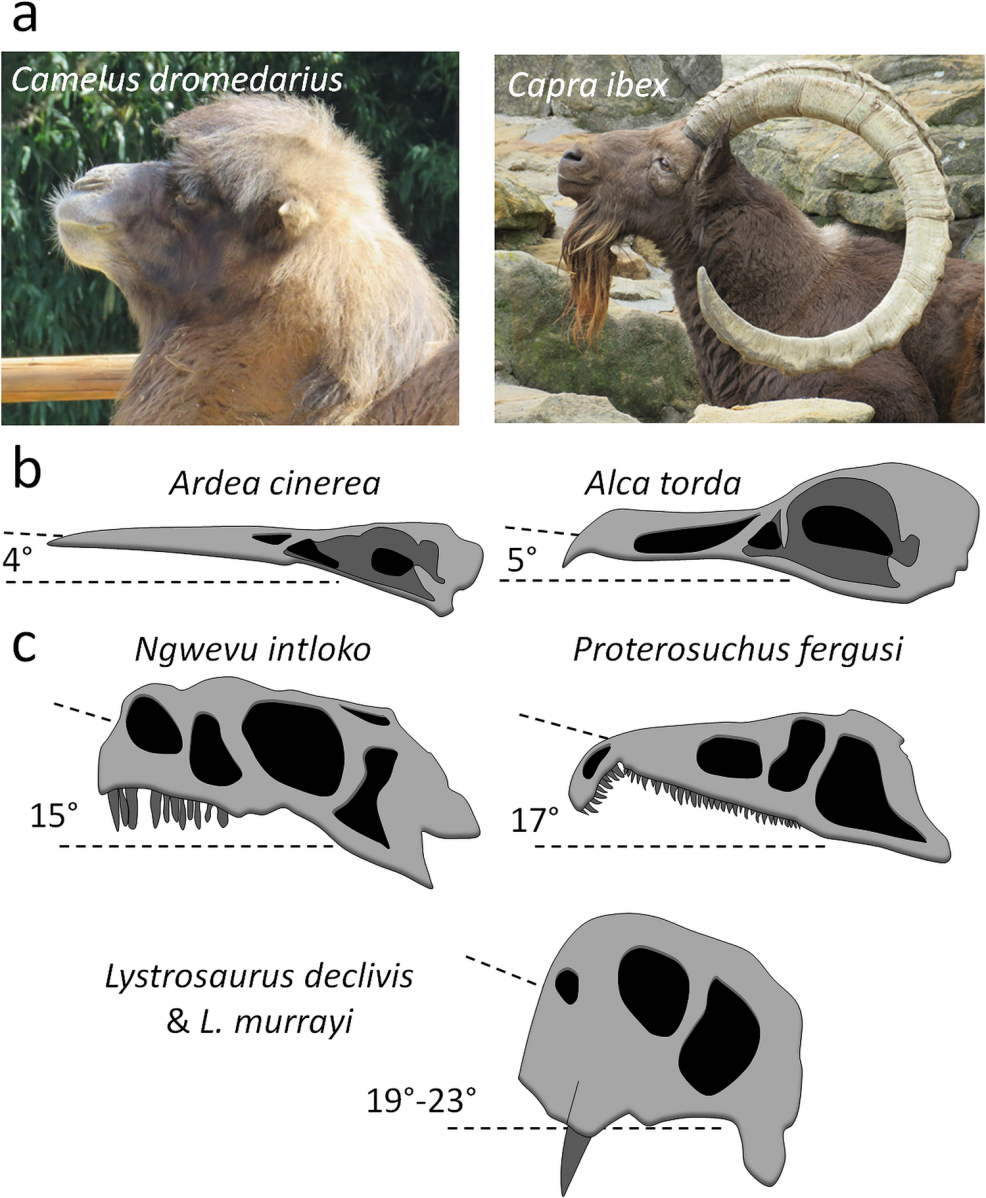
Examples of modern and extinct species in which the neutral or reconstructed head posture is tilted upward anteriorly. (**a**) The neutral head posture of two specimens of the tylopod *Camelus dromedarius* and caprin *Capra ibex*. (**b**) The reconstructed cranial orientation of two birds, *Ardea cinerea* and *Alca torda*, according to Duijm^8^.

No significant result indicative of a correlation between habitat and head posture or reconstructed cranial orientation was found; however, semi-aquatic species show a noticeably high reconstructed posture of the skull on average (23°) compared to other species (Fig. 6). The low number of semi-aquatic species in the dataset (one rhinocerotid, three tapirids, and two hippopotamids, Table 1) likely prevents this trend to be identified as significant in our sample. A high head posture is not observed in semi-aquatic species (Fig. 6), even though it would be expected of species that have to keep breathing above water level most of the time while immersed^27,30^. Recently, a semi-aquatic habit for the Triassic archosaur *Proterosuchus* and the therapsid *Lystrosaurus* has been hypothesized as these two species would have their head tilted upward anteriorly when the plane of the LSC is horizontal^27,94^ (Fig. 11c). In *Proterosuchus* this upward tilting would be about 17°^27^, whereas in *Lystrosaurus* it would be between 19° and 23°^94^. However, an upward tilting of the neutral head posture is also habitually observed in many fully terrestrial species, such as *Camelus dromedarius* and was occasionally spotted in *Capra ibex* (Fig. 11a; see Supplementary Table S1). Among modern archosaurs, an upward tilting of the head is observed in some sea birds and the common starling *Sturnus vulgaris*^8^. An upward tilting of the beak when the dry skull is held with the plane of the LSC horizontal seems to be observed in the razorbill (*Alca torda*) and the Heron (*Ardea cinerea*) (as inferred from the figures and measurements in Duijm^8^, see Supplementary Table S1); however, the reconstructed cranial orientations in the razorbill and heron vary between 0° and -4° only (Fig. 11b).

Among fossil species, the likely terrestrial sauropod *Ngwevu intloko* (Fig. 11c) would also have had its reconstructed cranial orientation tilted 17° above the horizontal (identified as *Massospondylus carinatus* in Sereno et al.^20^; see Chapelle et al.^95^). The peculiar orientation of the LSC in *Lystrosaurus*, *Proterosuchus*, and *Massospondylus* is remarkable as such a downward tilting of the LSC superior to 15° has never been found in any modern species to date, particularly not in the semi-aquatic species studied here which all have a posteriorly tilted LSC as all other “ungulates” (average reconstructed cranial orientation of semi-aquatic species = 23°) (Fig. 6; Table 1). The heron and razorbill mentioned above would have an anterior tilting of less than 4° (Supplementary Table S1), and Duijm’s^8^ dataset includes mostly semi-aquatic species, which limits comparisons. Overall, a correlation between an upward tilting of the LSC and semi-aquatic lifestyle is not supported by current data.

**Figure 12:**
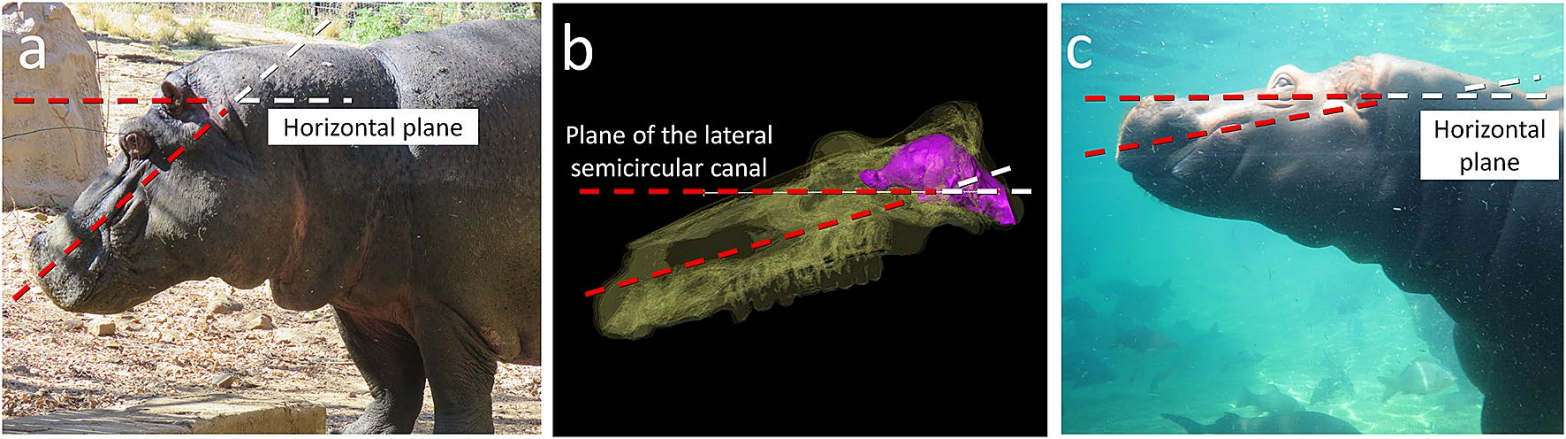
The neutral head posture (**a**), reconstructed cranial orientation (**b**), and head posture underwater (**c**) in *Hippopotamus amphibius*.

Noteworthily, the most aquatic species of the dataset, *Hippopotamus amphibius*, stands out on the scatter-plots as an outlier (Fig. 4). This is due to the large difference between its strongly anteriorly tilted neutral head posture and its almost horizontal reconstructed cranial orientation (difference = 39°). *Hippopotamus amphibius* normally spends very little time on land^49^, and it can be hypothesized that its bony labyrinth morphology would be more adapted to life in water than on land^96^. This hypothesis is supported by the orientation of the head while swimming in *H. amphibius*, which is more consistent with its reconstructed cranial orientation (Fig. 12), and the fact that the difference between the neutral and reconstructed head postures in the more terrestrial *Hexaprotodon liberiensis* (23°) falls more within the range of variation of “ungulates” (Fig. 4). Further field observations of underwater hippopotamus head posture will be necessary to address this hypothesis.

### The influence of head-butting

**Figure 13:**
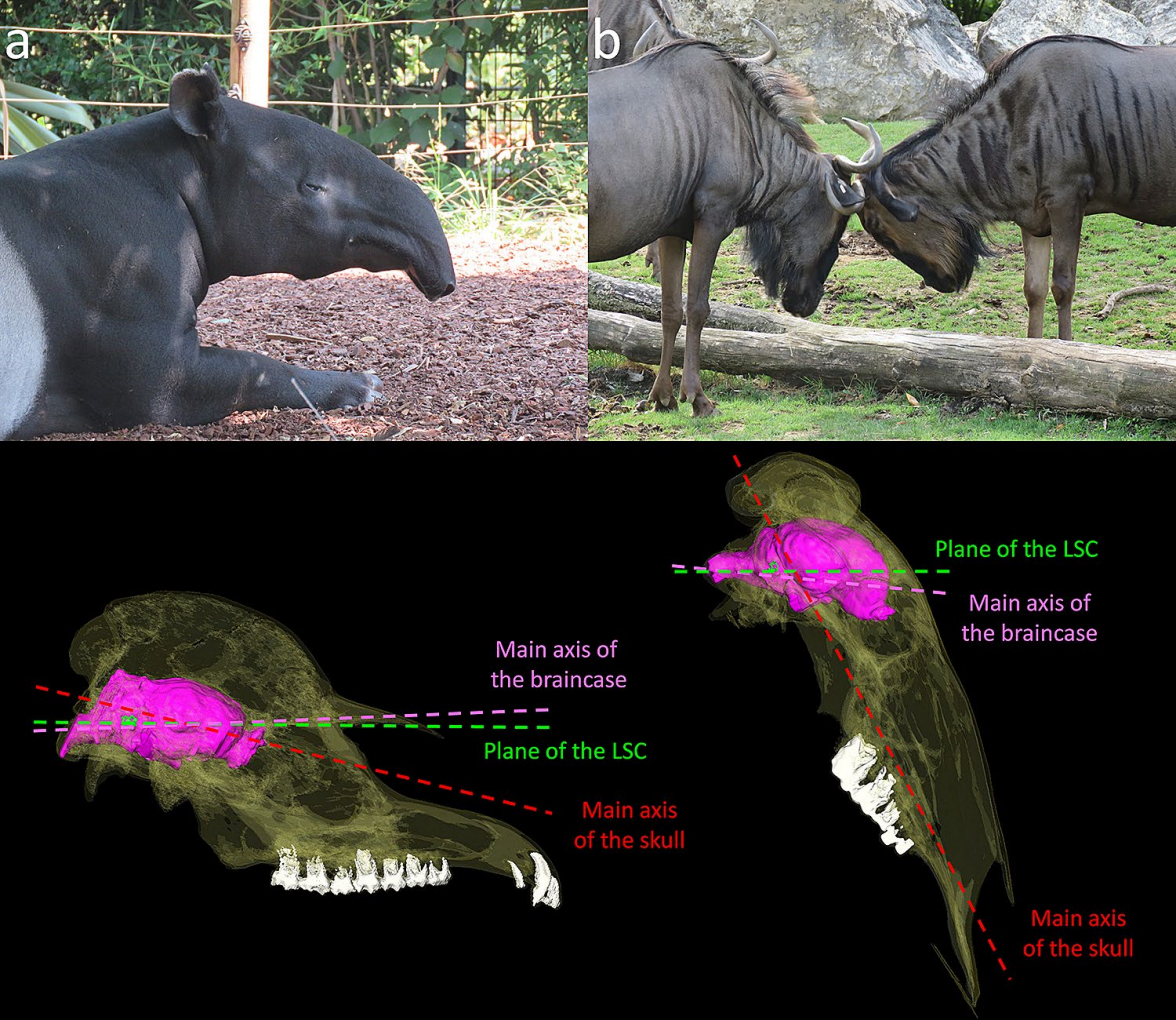
Comparison of the cranial (transparent), endocranial (pink), and LSC (green) orientations in a non-head-butting species (*Tapirus indicus*, **a**) and a head-butting species (*Connochaetes taurinus*, **b**).

**Figure 14:**
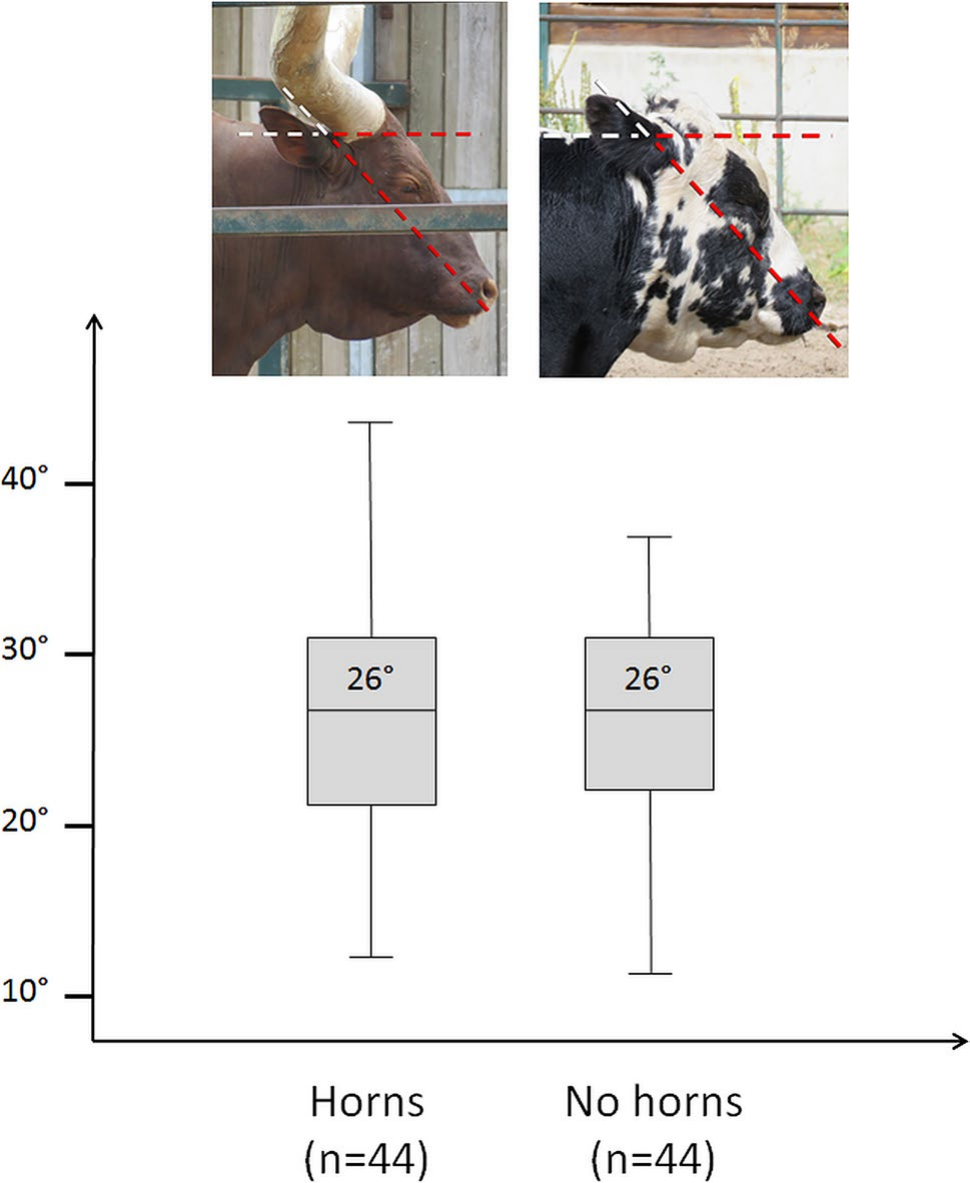
Boxplot of intraspecific variation of neutral head posture in degrees (°) in individuals in which horns, antlers, or ossicones are present or large (Horns) and absent or small (No horns). The average angle for each category is indicated in the corresponding boxplot. n represents the number of measurements in each category.

Taxa that engage in head-to-head combat have almost the same average neutral orientation of their head as non-head-butting taxa (34° and 33° respectively) (Fig. 7). In sharp contrast, they show a significantly more anteriorly tilted reconstructed cranial orientation (average = 46°) compared to non-head-butting taxa (average = 33°). Unlike what is observed between browsers and grazers, the values here are markedly different (Fig. 7) and the difference is highly statistically significant (p-value = 0.002). It is unlikely that the reconstructed cranial orientation reflects the posture during head-butting, as animals keep their head extremely low during this activity^49,92,93^, much lower than their corresponding reconstructed cranial orientation (Fig. 13b). A phenomenon of re-orientation of the braincase and basicranium in head-butting “ungulates” that would not affect head posture overall seems more probable. Such cranial flexure would result in a misalignment of the main axis of the braincase with that of the snout (Fig. 13), a condition termed cyptocephaly and commonly encountered in head-butting “ungulates”^33,48,92,97–99^. This implies that natural selection on the alignment of the plane of the LSC to the horizontal was muted by another, likely more important adaptation to head-butting. This may be the necessity to re-orientate the braincase and basicranium in order to align the fighting surface of the skull, occipital condyles, and vertebral column to help dissipate the energy of the impact to the body and away from the brain^24,100,101^. Another hypothesis would be that the re-organization of the braincase serves to accommodate the development of the large cranial apparatuses found in most head-butting species (e.g. horns and antlers)^29^. In our dataset, 56% of species without horns do not head-butt, and 83% of species with horns do head-butt (Supplementary Table S1), which would partly support this hypothesis. In contrast, intraspecific variations in the presence or absence of horns appear to have no impact on neutral head posture on average (Fig. 14; Supplementary Table S1). These two hypotheses will need further observations and biomechanical modeling to be addressed adequately.

## Concluding remarks

Neutral head posture is here found to be significantly correlated to the orientation of the plane of the LSC in “ungulate” mammals, but this relationship is loose, and it appears that diet and head-butting have an effect on LSC orientation although not on neutral head posture as would be expected. This suggests an overall relaxed constraint on the alignment of the plane of the LSC to the horizontal at rest. Head posture during locomotion and/or adaptation to head-butting might play a more significant role in the orientation of the LSC than its horizontality at rest, two possibilities that will have to be addressed further.

In this contribution, some noteworthy trends between the orientation of the LSC, body mass, diet, adaptation to a semi-aquatic environment, and head-butting are pointed out, although many of these ecological components are difficult to disentangle. “Ungulates” living in closed habitats are often smaller than in open habitats, more solitary, browsers and tend to fight for mates by stabbing each other with their horns and teeth, whereas species from more open habitats usually graze in large herds and perform head-butting to ascertain dominance and attract mates^34,36,40,47^.

Finally, although this study finds that there is some interesting ecological and behavioral signal in the orientation of the LSC of ungulates that could be exploited by paleontologists, it is crucial to highlight that the phylogenetic signal was highly significant for all the variables examined here and as such, what the orientation of the LSC reflects the best in “ungulates” is their phylogeny more than anything else. Further understanding of the evolutionary processes associated with such a strong phylogenetic disparity will require investigating each subclade in the sample individually and a more exhaustive sample for each of them.

## Supplementary information

Supplementary Information

Supplementary Information

## Data Availability

The datasets analysed during the current study are available in the Supplementary Table S1 and online at this URL: https://osf.io/4vpnj/?view_only=3dc987012fcd44a6a64ad7d8949ec01f (10.17605/Osf.Io/4vpnj). Data replicated in the Wits Data Archive and can be guaranteed to be in the holding of the University of Witwatersrand.

## References

[1] Schoenemann, A. *Schläfenbein und Schädelbasis, eine anatomisch-otiatrische Studie*. (Gesellsch., 1906).

[2] Benoit-Gonin L, Lafite-Dupont P (1907). Destinée du canal semi-circulaire externe dans le passage de la station quadrupède à la station bipède. C R Soc. Biol. Paris.

[3] Girard LL (1923). plan des canaux semi-circulaires horizontaux considéré comme plan horizontal de la tête. Bull. Mem. Soc. Anthropol. Paris Ser..

[4] Ledebkin L (1924). Über die Lage des Canalis semicircularis lateralis bei Säugern. Anatonzischer Anzeiger.

[5] Girard L (1929). L’attitude normale de la tête déterminée par le labyrinthe de l’oreille. Bull. Mem. Soc. Anthropol. Paris Serie.

[6] de Beer GR (1947). How animals hold their heads. Proc. Linn Soc. Lond..

[7] Van Der Klaauw CJ (1948). Size and position of the functional components of the skull: a contribution to the knowledge of the architecture of the skull, based on data in the literature. Arch. Néerl. Zool..

[8] Duijm MJ (1951). On the head posture in birds and its relation features. Proceedings of the Koninklijke Nederlandse Akademie van Wetenschappen C.

[9] Saban, R. Fixité du canal-semicirculaire externe et variations de l’angle thyridien. *Mammalia* **16**, (1952).

[10] Anthony, J. Justification des principes de la méthode véstibulaire. *Mammalia* **17**, (1953).

[11] Vidal PP, Graf W, Berthoz A (1986). The orientation of the cervical vertebral column in unrestrained awake animals I. Resting position. Exp. Brain Res..

[12] Hullar TE (2006). Semicircular canal geometry, afferent sensitivity, and animal behavior. Anat. Rec..

[13] Graf W, Klam FL (2006). système vestibulaire : anatomie fonctionnelle et comparée, évolution et développement. C R Palevol.

[14] Fitzpatrick RC, Butler JE, Day BL (2006). Resolving Head Rotation for Human Bipedalism. Curr Biol.

[15] Cox PG, Jeffery N (2010). Semicircular canals and agility: the influence of size and shape measures. J Anat.

[16] Malinzak MD, Kay RF, Hullar TE (2012). Locomotor head movements and semicircular canal morphology in primates. PNAS.

[17] Hotton, N. I. Dicynodonts and their role as primary consumers. in *The Ecology and Biology of Mammal-like Reptiles* (eds. Hotton, N. I., MacLean, P. D., Roth, J. J. & Roth, E. C.) 71–82 (Smithsonian Institution Press, 1986).

[18] Spoor F, Wood B, Zonneveld F (1994). Implications of early hominid labyrinthine morphology for evolution of human bipedal locomotion. Nature.

[19] Witmer LM, Chatterjee S, Franzosa J, Rowe T (2003). Neuroanatomy of flying reptiles and implications for flight, posture and behaviour. Nature.

[20] Sereno PC (2007). Structural extremes in a cretaceous dinosaur. PLoS ONE.

[21] Witmer, L. M., Ridgely, R. C., Dufeau, D. L. & Semones, M. C. Using CT to Peer into the Past: 3D Visualization of the Brain and Ear Regions of Birds, Crocodiles, and Nonavian Dinosaurs. in *Anatomical Imaging* (eds. Endo, H. & Frey, R.) 67–87 (Springer Japan, 2008). doi:10.1007/978-4-431-76933-0_6.

[22] Witmer LM, Ridgely RC (2009). New insights into the brain, braincase, and ear region of tyrannosaurs (Dinosauria, Theropoda), with implications for sensory organization and behavior. Anat Rec.

[23] Badlangana NL, Adams JW, Manger PR (2011). A comparative assessment of the size of the frontal air sinus in the Giraffe (*Giraffa camelopardalis*). Anat. Rec..

[24] Benoit J, Manger PR, Norton L, Fernandez V, Rubidge BS (2017). Synchrotron scanning reveals the palaeoneurology of the head-butting *Moschops capensis* (Therapsida, Dinocephalia). PeerJ.

[25] Araújo R, Fernandez V, Polcyn MJ, Fröbisch J, Martins RMS (2017). Aspects of gorgonopsian paleobiology and evolution: insights from the basicranium, occiput, osseous labyrinth, vasculature, and neuroanatomy. PeerJ.

[26] Bullar CM, Zhao Q, Benton MJ, Ryan MJ (2019). Ontogenetic braincase development in *Psittacosaurus lujiatunensis* (Dinosauria: Ceratopsia) using micro-computed tomography. PeerJ.

[27] Brown EE, Butler RJ, Ezcurra MD, Bhullar B-AS, Lautenschlager S (2020). Endocranial anatomy and life habits of the Early Triassic archosauriform *Proterosuchus fergusi*. Palaeontol.

[28] Schade M, Rauhut OWM, Evers SW (2020). Neuroanatomy of the spinosaurid *Irritator challengeri* (Dinosauria: Theropoda) indicates potential adaptations for piscivory. Sci. Rep..

[29] Sakagami R, Kawabe S (2020). Endocranial anatomy of the ceratopsid dinosaur *Triceratops* and interpretations of sensory and motor function. PeerJ.

[30] Schellhorn R (2018). A potential link between lateral semicircular canal orientation, head posture, and dietary habits in extant rhinos (Perissodactyla, Rhinocerotidae). J. Morphol..

[31] Neenan JM, Scheyer TM (2012). The braincase and inner ear of *Placodus gigas* (Sauropterygia, Placodontia)—a new reconstruction based on micro-computed tomographic data. J. Vert. Paleontol..

[32] Seymour RS (2016). Cardiovascular physiology of dinosaurs. Physiology.

[33] Schaffer, W. M. & Reed, C. A. *The co-evolution of social behavior and cranial morphology in sheep and goats (Bovidae, caprini)*. 1–112 (Field Museum of Natural History, 1972). doi:10.5962/bhl.title.2828.

[34] Geist V (1966). The evolution of horn-like organs. Behav.

[35] Pérez-Barbería FJ, Gordon IJ (2005). Gregariousness increases brain size in ungulates. Oecologia.

[36] Emlen DJ (2008). The evolution of animal weapons. Annu. Rev. Ecol. Evol. Syst..

[37] Boucot, A. J. & Poinar, G. O. Jr. *Fossil Behavior Compendium*. (CRC Press, 2011).

[38] Snively E, Theodor JM (2011). Common functional correlates of head-strike behavior in the pachycephalosaur *Stegoceras validum* (Ornithischia, Dinosauria) and combative artiodactyls. PLoS ONE.

[39] Peterson JE, Vittore CP (2012). Cranial pathologies in a specimen of *Pachycephalosaurus*. PLoS ONE.

[40] Cabrera D, Stankowich T (2018). Stabbing slinkers: tusk evolution among artiodactyls. J. Mammal. Evol..

[41] Brichta AM, Acuña DL, Peterson EH (1988). Planar relations of semicircular canals in awake, resting turtles *Pseudemys scripta*. BBE.

[42] Boistel R (2010). Assisted walking in Malagasy dwarf chamaeleons. Biol. Lett..

[43] Marugán-Lobón J, Chiappe LM, Farke AA (2013). The variability of inner ear orientation in saurischian dinosaurs: testing the use of semicircular canals as a reference system for comparative anatomy. PeerJ.

[44] Taylor MP, Wedel MJ, Naish D (2009). Head and neck posture in sauropod dinosaurs inferred from extant animals. Acta Palaeontol. Pol..

[45] Coutier F, Hautier L, Cornette R, Amson E, Billet G (2017). Orientation of the lateral semicircular canal in Xenarthra and its links with head posture and phylogeny. J. Morphol..

[46] Caix M, Outrequin GL (1979). variabilité des canaux semi-circulaires osseux. Anat. Clin.

[47] Köhler, M. *Skeleton and Habitat of Recent and Fossil Ruminants*. (F. Pfeil, 1993).

[48] Solounias, N. Family Bovidae. in *The Evolution of Artiodactyls* (eds. Prothero, D. R. & Foss, S. E.) 278–291 (University of The Johns Hopkins Press, 2007).

[49] Estes, R. D. *The Behavior Guide to African Mammals: Including Hoofed Mammals, Carnivores, Primates*. (Univ of California Press, 2012).

[50] Mennecart B, Costeur L (2016). Shape variation and ontogeny of the ruminant bony labyrinth, an example in Tragulidae. J. Anat..

[51] Costeur L, Mennecart B, Müller B, Schulz G (2017). Prenatal growth stages show the development of the ruminant bony labyrinth and petrosal bone. J. Anat..

[52] Lyu H-Y (2016). The age-related orientational changes of human semicircular canals. Clin. Exp. Otorhinolaryngol..

[53] Neenan JM, Chapelle KEJ, Fernandez V, Choiniere JN (2019). Ontogeny of the *Massospondylus* labyrinth: implications for locomotory shifts in a basal sauropodomorph dinosaur. Palaeontology.

[54] Felsenstein J (1985). Phylogenies and the comparative method. Am. Nat..

[55] Paradis, E. *Analysis of Phylogenetics and Evolution with R*. (Springer New York, 2012). doi:10.1007/978-1-4614-1743-9.

[56] *Modern phylogenetic comparative methods and their application in evolutionary biology: concepts and practice*. (Springer Berlin Heidelberg, 2014). doi:10.1007/978-3-662-43550-2.

[57] Bininda-Emonds ORP (2007). The delayed rise of present-day mammals. Nature.

[58] Paradis, E. & Schliep, K. ape 5.0: an environment for modern phylogenetics and evolutionary analyses in R. *Bioinformatics* **35**, 526–528 (2019). 10.1093/bioinformatics/bty633 30016406

[59] Pagel M (1999). Inferring the historical patterns of biological evolution. Nature.

[60] Revell LJ (2012). phytools: an R package for phylogenetic comparative biology (and other things). Methods Ecol. Evol..

[61] Blomberg SP, Garland T, Ives AR (2003). Testing for phylogenetic signal in comparative data: behavioral traits are more labile. Evolution.

[62] Münkemüller T (2012). How to measure and test phylogenetic signal. Methods Ecol. Evol..

[63] Molina-Venegas R, Rodríguez MÁ (2017). Revisiting phylogenetic signal; strong or negligible impacts of polytomies and branch length information?. BMC Evol. Biol..

[64] Fritz SA, Purvis A (2010). Selectivity in mammalian extinction risk and threat types: a new measure of phylogenetic signal strength in binary traits. Conserv. Biol..

[65] Orme D (2013). CAPER: comparative analyses of phylogenetics and evolution in R. Methods Ecol. Evol..

[66] Grafen A, Hamilton WD (1989). The phylogenetic regression. Philos. Trans. R Soc. Lond. B Biol. Sci..

[67] Symonds, M. R. E. & Blomberg, S. P. A primer on phylogenetic generalised least squares. In *Modern Phylogenetic Comparative Methods and Their Application in Evolutionary Biology: Concepts and Practice* (ed. Garamszegi, L. Z.) 105–130 (Springer, 2014). doi:10.1007/978-3-662-43550-2_5.

[68] Pinheiro, J. C., Bates, D. J., DebRoy, S. & Sakar, D. *The Nlme package: linear and nonlinear mixed effects models, R Version 3*. *R package version* vol. 6 (2012).

[69] Mazerolle, M. *Model Selection and Multimodel Inference Based on (Q)AIC(c)*. *R package version* vol. 2 (2013).

[70] Benoit J (2019). Brain evolution in Proboscidea (Mammalia, Afrotheria) across the Cenozoic. Sci. Rep..

[71] Jerison, H. *Evolution of The Brain and Intelligence.* (Elsevier Science, 1973).

[72] Spoor F (2007). The primate semicircular canal system and locomotion. PNAS.

[73] Spoor F, Bajpai S, Hussain ST, Kumar K, Thewissen JGM (2002). Vestibular evidence for the evolution of aquatic behaviour in early cetaceans. Nature.

[74] Veall MR, Zimmermann KF (1996). Pseudo-R2 measures for some common limited dependent variable models. J. Econ. Surveys.

[75] Mundry, R. Statistical issues and assumptions of phylogenetic generalized least squares. in *Modern Phylogenetic Comparative Methods and Their Application in Evolutionary Biology: Concepts and Practice* (ed. Garamszegi, L. Z.) 131–153 (Springer, 2014). doi:10.1007/978-3-662-43550-2_6.

[76] Garland T, Dickerman AW, Janis CM, Jones JA (1993). Phylogenetic analysis of covariance by computer simulation. Syst. Biol..

[77] Benjamini, Y. & Hochberg, Y. Controlling the false discovery rate: a practical and powerful approach to multiple testing. *J. R. Stat. Soc. Ser. B (Methodological)* **57**, 289–300 (1995).

[78] Harmon LJ (2010). Early bursts of body size and shape evolution are rare in comparative data. Evolution.

[79] Romano, M., Manucci, F. & Palombo, M. R. The smallest of the largest: new volumetric body mass estimate and in-vivo restoration of the dwarf elephant *Palaeoloxodon* ex gr. *P. falconeri* from Spinagallo Cave (Sicily). *Hist Biol* **1**, 1–14 (2019).

[80] Romano, M. & Manucci, F. Resizing Lisowicia bojani: volumetric body mass estimate and 3D reconstruction of the giant Late Triassic dicynodont. *Hist Biol* **0**, 1–6 (2019).

[81] Campione, N. E. & Evans, D. C. The accuracy and precision of body mass estimation in non‐avian dinosaurs. *Biol. Rev. brv.* 12638 (2020). doi:10.1111/brv.12638. 10.1111/brv.12638 32869488

[82] Hopkins, S. S. B. Estimation of Body Size in Fossil Mammals. in *Methods in Paleoecology: Reconstructing Cenozoic Terrestrial Environments and Ecological Communities* (eds. Croft, D. A., Su, D. F. & Simpson, S. W.) 7–22 (Springer International Publishing, 2018). doi:10.1007/978-3-319-94265-0_2.

[83] Spoor F, Zonneveld F (1998). Comparative review of the human bony labyrinth. Am. J. Phys. Anthropol..

[84] Dunbar DC, Macpherson JM, Simmons RW, Zarcades A (2008). Stabilization and mobility of the head, neck and trunk in horses during overground locomotion: comparisons with humans and other primates. J. Exp. Biol..

[85] Zubair HN, Beloozerova IN, Sun H, Marlinski V (2016). Head movement during walking in the cat. Neuroscience.

[86] Mennecart B, Costeur L (2016). A *Dorcatherium* (Mammalia, Ruminantia, Middle Miocene) petrosal bone and the tragulid ear region. J. Vert. Paleontol..

[87] Mennecart B (2017). Bony labyrinth morphology clarifies the origin and evolution of deer. Sci. Rep..

[88] Aiglstorfer M, Costeur L, Mennecart B, Heizmann EPJ (2017). *Micromeryx? eiselei*—a new moschid species from Steinheim am Albuch, Germany, and the first comprehensive description of moschid cranial material from the Miocene of Central Europe. PLoS ONE.

[89] Prothero, D. R. & Schoch, R. M. *Horns, Tusks, and Flippers: The Evolution of Hoofed Mammals*. (JHU Press, 2002).

[90] Ackerly D (2009). Conservatism and diversification of plant functional traits: evolutionary rates versus phylogenetic signal. PNAS.

[91] Cadotte MW, Davies TJ, Peres-Neto PR (2017). Why phylogenies do not always predict ecological differences. Ecol. Monogr..

[92] Geist, V. *Deer of the world: their evolution, behaviour, and ecology*. (Stackpole Books, 1998).

[93] Walker, E. P., Nowak, R. M. & Paradiso, J. L. *Walker’s Mammals of the world*. (Johns Hopkins University Press, 1983).

[94] Benoit J, Manger PR, Fernandez V, Rubidge BS (2017). The bony labyrinth of late Permian Biarmosuchia: palaeobiology and diversity in non-mammalian Therapsida. Palaeontol. Afr..

[95] Chapelle KEJ, Barrett PM, Botha J, Choiniere JN (2019). Ngwevu intloko: a new early sauropodomorph dinosaur from the Lower Jurassic Elliot Formation of South Africa and comments on cranial ontogeny in *Massospondylus carinatus*. Peer J..

[96] Mourlam MJ, Orliac MJ (2017). Infrasonic and ultrasonic hearing evolved after the emergence of modern whales. Curr. Biol..

[97] Osborn, H. F. *The titanotheres of ancient Wyoming, Dakota, and Nebraska*. (Washington [D.C.] : Dept. of the Interior, U.S. Geological Survey, 1929).

[98] Osborn, H. F. *Proboscidea : a monograph of the discovery, evolution, migration and extinction of the mastodonts and elephants of the world*. vol. I (New York : Published on the J. Pierpont Morgan Fund by the trustees of the American Museum of Natural History : American Museum Press, 1936).

[99] Osborn, H. F. *Proboscidea : a monograph of the discovery, evolution, migration and extinction of the mastodonts and elephants of the world*. vol. II (New York : Published on the J. Pierpont Morgan Fund by the trustees of the American Museum of Natural History : American Museum Press, 1942).

[100] Stanley SM (1974). Relative growth of the titanothere horn: a new approach to an old problem. Evolution.

[101] Barghusen HR (1975). A review of fighting adaptations in dinocephalians (Reptilia, Therapsida). Paleobiol.

