# Supplementary material for: A test of the lateral semicircular canal correlation to head posture, diet and other biological traits in “ungulate” mammals
Source: Sci Rep. 2020 Nov 11;10:19602. doi: 10.1038/s41598-020-76757-0 (PMC7658238; doi:10.1038/s41598-020-76757-0)
Supplement: Supplementary file 1 — Supplementary Information [file 41598_2020_76757_MOESM1_ESM.pdf]

A test of the lateral semicircular canal correlation to head posture, diet and other biological traits in "ungulate" mammals.

Benoit J. [1]*, Legendre L.J. [2], Farke A.A.[3], Neenan J.M. [4], Mennecart B. [5], Costeur L. [5], Merigeaud S. [6], Manger P.R.[7]

[1]Evolutionary Studies Institute (ESI), School of Geosciences, University of the Witwatersrand, Braamfontein, 2050, Johannesburg, South Africa

[2]Jackson School of Geosciences, The University of Texas at Austin, 2275 Speedway Stop C9000, Austin, TX, United States

[3] Raymond M. Alf Museum of Paleontology at The Webb Schools, 1175 West Baseline Road, Claremont, California, USA

[4] Oxford University Museum of Natural History, Parks Road, Oxford, OX1 3PW, UK.

[5] Naturhistorisches Museum Basel, Augustinergasse 2, 4001 Basel, Switzerland.

[6] Radiologist Groupe CRP, Clinique du Parc, Castelnau le Lez, France

Tridilogy, Saint Gely du Fesc, France

[7] School of Anatomical Sciences, University of the Witwatersrand, 7 York Road, Parktown, 2193, Johannesburg, South Africa.

*Corresponding author: julien.benoit@wits.ac.za.

Supplementary Figures

Figure S1. Ancestral character state reconstruction of the neutral head posture in ungulates using maximum likelihood (fastAnc) in phytools (Revell, 2013). Phylogenetic tree after Bininda-Emonds et al. [56]

Figure S2. Ancestral character state reconstruction of of the reconstructed head posture in ungulates using maximum likelihood (fastAnc) in phytools (Revell, 2013). Phylogenetic tree after Bininda-Emonds et al. [56]

Figure S3. Ancestral character state reconstruction of body mass variations in ungulates using MCMC stochastic character mapping (make.simmap) in phytools (Revell, 2013). a, continuous data; b, character discretized into a binary data (<100 kg/>100 kg). Phylogenetic tree after Bininda-Emonds et al. [56]

Figure S4. Ancestral character state reconstruction of diet variations in ungulates using MCMC stochastic character mapping (make.simmap) in phytools (Revell, 2013). Phylogenetic tree after Bininda-Emonds et al. [56]

Figure S5. Ancestral character state reconstruction of the habitats variations in ungulates using MCMC stochastic character mapping (make.simmap) in phytools (Revell, 2013). Phylogenetic tree after Bininda-Emonds et al. [56]

Figure S6. Ancestral character state reconstruction of head butting behaviour in ungulates using MCMC stochastic character mapping (make.simmap) in phytools (Revell, 2013). Phylogenetic tree after Bininda-Emonds et al. [56]

Reference list:

Revell, L.J., 2013. Two new graphical methods for mapping trait evolution on phylogenies. Methods Ecol Evol 4, 754–759. https://doi.org/10.1111/2041-210X.12066

Fig. S1

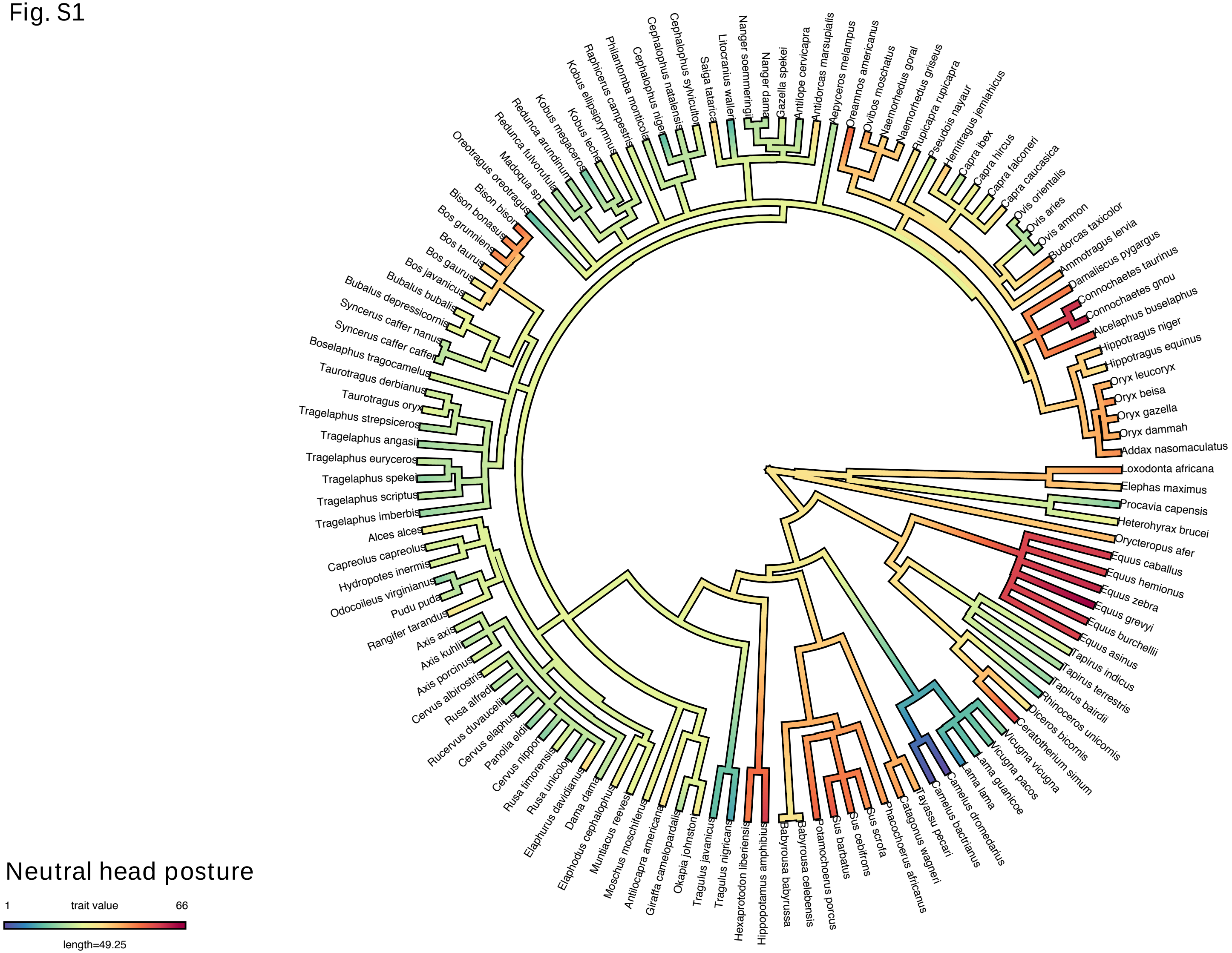
Oreotragus oreotragus
Madoqua sp.
Redunca fulvorufula
Redunca arundinum
Kobus megaceros
Kobus leche
Kobus ellipsiprymnus
Raphicerus campestris
Philantomba monticola
Cephalophus niger
Cephalophus natalensis
Cephalophus sylvicultor
Saiga tatarica
Litocranius walleri
Nanger soemmeringii
Nanger dama
Gazella spekei
Antilope cervicapra
Antidorcas marsupialis
Aepyceros melampus
Oreamnos americanus
Ovibos moschatus
Naemorhedus goral
Naemorhedus griseus
Rupicapra rupicapra
Pseudois nayaur
Hemitragus jemlahicus
Capra ibex
Capra hircus
Capra falconeri
Capra caucasica
Ovis orientalis
Ovis aries
Ovis ammon
Budorcas taxicolor
Ammotragus lervia
Damaliscus pygargus
Connochaetes taurinus
Connochaetes gnou
Alcelaphus buselaphus
Hippotragus niger
Hippotragus equinus
Oryx leucoryx
Oryx beisa
Oryx gazella
Oryx dammah
Addax nasomaculatus
Loxodonta africana
Elephas maximus
Procavia capensis
Heterohyrax brucei
Orycteropus afer
Equus caballus
Equus hemionus
Equus zebra
Equus grevyi
Equus burchellii
Equus asinus
Tapirus indicus
Tapirus terrestris
Tapirus bairdii
Rhinoceros unicornis
Diceros bicornis
Ceratotherium simum
Vicugna vicugna
Vicugna pacos
Lama guanicoe
Lama lama
Camelus dromedarius
Camelus bactrianus
Tayassu pecari
Catagonus wagneri
Phacochoerus africanus
Sus scrofa
Sus cebifrons
Sus barbatus
Potamochoerus porcus
Babyrousa celebensis
Babyrousa babyrussa
Hippopotamus amphibius
Hexaprotodon liberiensis
Tragulus nigricans
Tragulus javanicus
Okapia johnstoni
Giraffa camelopardalis
Antilocapra americana
Moschus moschiferus
Muntiacus reevesi
Elaphodus cephalophus
Dama dama
Elaphurus davidianus
Rusa unicolor
Rusa timorensis
Cervus nippon
Panolia eldi
Cervus elaphus
Rucervus duvaucelii
Rusa alfredi
Cervus albirostris
Axis porcinus
Axis kuhlii
Axis axis
Rangifer tarandus
Pudu puda
Odocoileus virginianus
Hydropotes inermis
Capreolus capreolus
Alces alces
Tragelaphus imberbis
Tragelaphus scriptus
Tragelaphus spekei
Tragelaphus euryceros
Tragelaphus angasii
Tragelaphus strepsiceros
Taurotragus oryx
Taurotragus derbianus
Boselaphus tragocamelus
Syncerus caffer caffer
Syncerus caffer nanus
Bubalus depressicornis
Bubalus bubalis
Bos javanicus
Bos gaurus
Bos taurus
Bos grunniens
Bison bonasus
Bison bison
Neutral head posture
1
trait value
66
length=49.25

Fig. S2

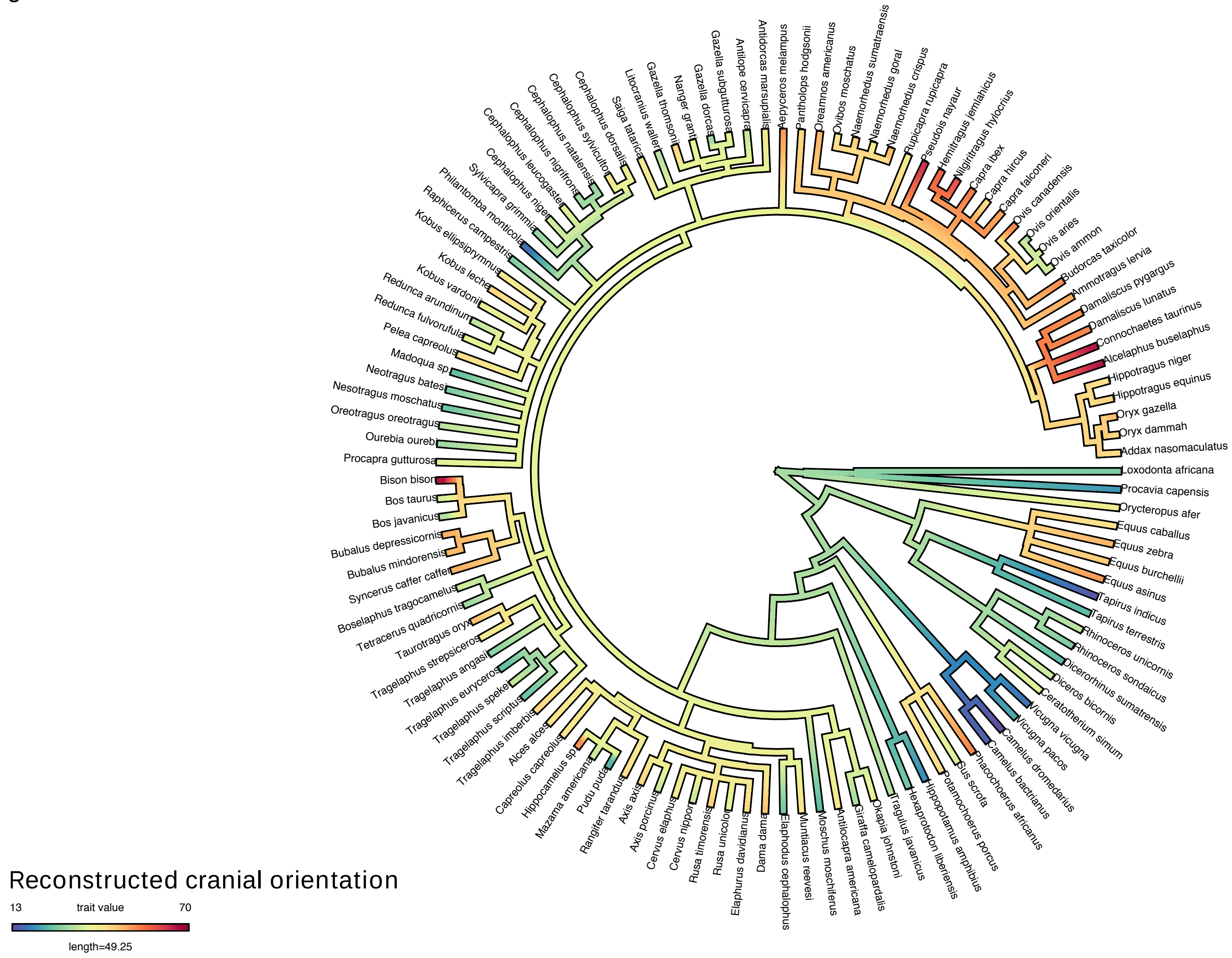
Antidorcas marsupialis
Antilope cervicapra
Gazella subgutturosa
Gazella dorcas
Nanger granti
Gazella thomsonii
Litocranius walleri
Saiga tatarica
Cephalophus dorsalis
Cephalophus sylvicultor
Cephalophus natalensis
Cephalophus nigrifrons
Cephalophus leucogaster
Cephalophus niger
Sylvicapra grimmia
Philantomba monticola
Raphicerus campestris
Kobus ellipsiprymnus
Kobus leche
Kobus vardonii
Redunca arundinum
Redunca fulvorufula
Pelea capreolus
Madoqua sp.
Neotragus batesi
Nesotragus moschatus
Oreotragus oreotragus
Ourebia ourebi
Procapra gutturosa
Bison bison
Bos taurus
Bos javanicus
Bubalus depressicornis
Bubalus mindorensis
Syncerus caffer caffer
Boselaphus tragocamelus
Tetracerus quadricornis
Taurotragus oryx
Tragelaphus strepsiceros
Tragelaphus angasii
Tragelaphus euryceros
Tragelaphus spekei
Tragelaphus scriptus
Tragelaphus imberbis
Alces alces
Capreolus capreolus
Hippocamelus sp.
Mazama americana
Pudu puda
Rangifer tarandus
Axis axis
Axis porcinus
Cervus elaphus
Cervus nippon
Rusa timorensis
Rusa unicolor
Elaphurus davidianus
Dama dama
Elaphodus cephalophus
Muntiacus reevesi
Moschus moschiferus
Antilocapra americana
Giraffa camelopardalis
Okapia johnstoni
Tragulus javanicus
Hexaprotodon liberiensis
Hippopotamus amphibius
Potamochoerus porcus
Sus scrofa
Phacochoerus africanus
Camelus bactrianus
Camelus dromedarius
Vicugna pacos
Vicugna vicugna
Ceratotherium simum
Diceros bicornis
Dicerorhinus sumatrensis
Rhinoceros sondaicus
Rhinoceros unicornis
Tapirus terrestris
Tapirus indicus
Equus asinus
Equus burchellii
Equus zebra
Equus caballus
Orycteropus afer
Procavia capensis
Loxodonta africana
Addax nasomaculatus
Oryx dammah
Oryx gazella
Hippotragus equinus
Hippotragus niger
Alcelaphus buselaphus
Connochaetes taurinus
Damaliscus lunatus
Damaliscus pygargus
Ammotragus lervia
Budorcas taxicolor
Ovis ammon
Ovis aries
Ovis orientalis
Ovis canadensis
Capra falconeri
Capra hircus
Capra ibex
Nilgiritragus hylocrius
Hemitragus jemlahicus
Pseudois nayaur
Rupicapra rupicapra
Naemorhedus crispus
Naemorhedus goral
Naemorhedus sumatraensis
Ovibos moschatus
Oreamnos americanus
Pantholops hodgsonii
Aepyceros melampus
Reconstructed cranial orientation
13
trait value
70
length=49.25

Fig. S3a

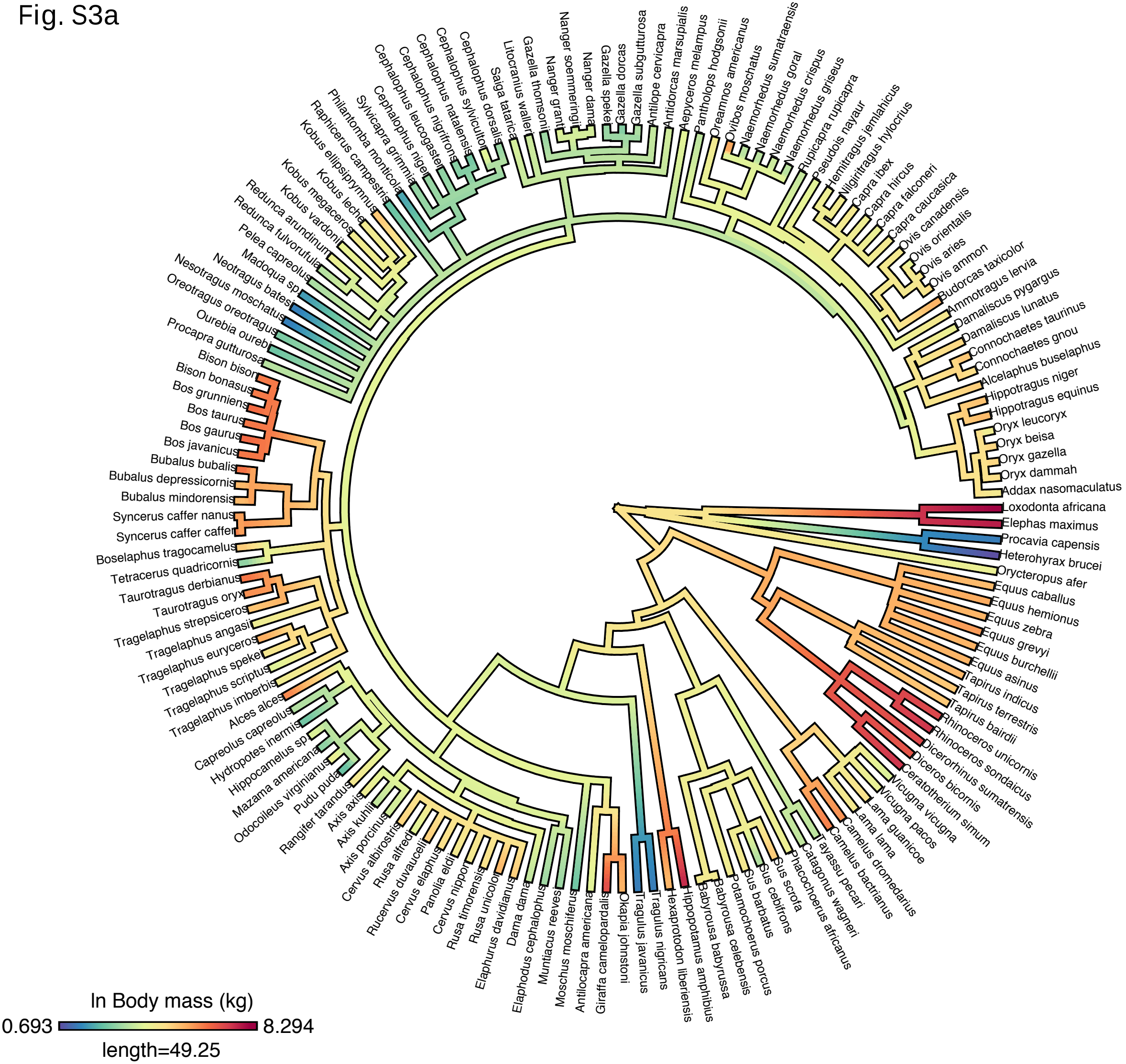

Procapra gutturosa
Ourebia ourebi
Oreotragus oreotragus
Nesotragus moschatus
Neotragus batesi
Madoqua sp
Pelea capreolus
Redunca fulvorufula
Redunca arundinum
Kobus vardonii
Kobus megaceros
Kobus leche
Kobus ellipsiprymnus
Raphicerus campestris
Philantomba monticola
Sylvicapra grimmia
Cephalophus niger
Cephalophus leucogaster
Cephalophus nigrifrons
Cephalophus natalensis
Cephalophus sylvicultor
Cephalophus dorsalis
Saiga tatarica
Litocranius walleri
Gazella thomsonii
Nanger granti
Nanger soemmeringii
Nanger dama
Gazella spekei
Gazella dorcas
Gazella subgutturosa
Antilope cervicapra
Antidorcas marsupialis
Aepyceros melampus
Pantholops hodgsonii
Oreamnos americanus
Ovibos moschatus
Naemorhedus sumatraensis
Naemorhedus goral
Naemorhedus crispus
Naemorhedus griseus
Rupicapra rupicapra
Pseudois nayaur
Hemitragus jemlahicus
Nilgiritragus hylocrius
Capra ibex
Capra hircus
Capra falconeri
Capra caucasica
Ovis canadensis
Ovis orientalis
Ovis aries
Ovis ammon
Budorcas taxicolor
Ammotragus lervia
Damaliscus pygargus
Damaliscus lunatus
Connochaetes taurinus
Connochaetes gnou
Alcelaphus buselaphus
Hippotragus niger
Hippotragus equinus
Oryx leucoryx
Oryx beisa
Oryx gazella
Oryx dammah
Addax nasomaculatus
Loxodonta africana
Elephas maximus
Procavia capensis
Heterohyrax brucei
Orycteropus afer
Equus caballus
Equus hemionus
Equus zebra
Equus grevyi
Equus burchellii
Equus asinus
Tapirus indicus
Tapirus terrestris
Tapirus bairdii
Rhinoceros unicornis
Rhinoceros sondaicus
Dicerorhinus sumatrensis
Diceros bicornis
Ceratotherium simum
Vicugna vicugna
Vicugna pacos
Lama guanicoe
Lama lama
Camelus dromedarius
Camelus bactrianus
Tayassu pecari
Catagonus wagneri
Phacochoerus africanus
Sus scrofa
Sus cebifrons
Sus barbatus
Potamochoerus porcus
Babyrousa celebensis
Babyrousa babyrussa
Hippopotamus amphibius
Hexaprotodon liberiensis
Tragulus nigricans
Tragulus javanicus
Okapia johnstoni
Giraffa camelopardalis
Antilocapra americana
Moschus moschiferus
Muntiacus reevesi
Elaphodus cephalophus
Dama dama
Elaphurus davidianus
Rusa unicolor
Rusa timorensis
Cervus nippon
Panolia eldii
Cervus elaphus
Rucervus duvaucelii
Rusa alfredi
Cervus albirostris
Axis porcinus
Axis kuhlii
Axis axis
Rangifer tarandus
Pudu puda
Odocoileus virginianus
Mazama americana
Hippocamelus sp
Hydropotes inermis
Capreolus capreolus
Alces alces
Tragelaphus imberbis
Tragelaphus scriptus
Tragelaphus spekei
Tragelaphus eurycerus
Tragelaphus angasii
Tragelaphus strepsiceros
Taurotragus oryx
Taurotragus derbianus
Tetracerus quadricornis
Boselaphus tragocamelus
Syncerus caffer caffer
Syncerus caffer nanus
Bubalus mindorensis
Bubalus depressicornis
Bubalus bubalis
Bos javanicus
Bos gaurus
Bos taurus
Bos grunniens
Bison bonasus
Bison bison
ln Body mass (kg)
0.693
8.294
length=49.25

Fig. S3b

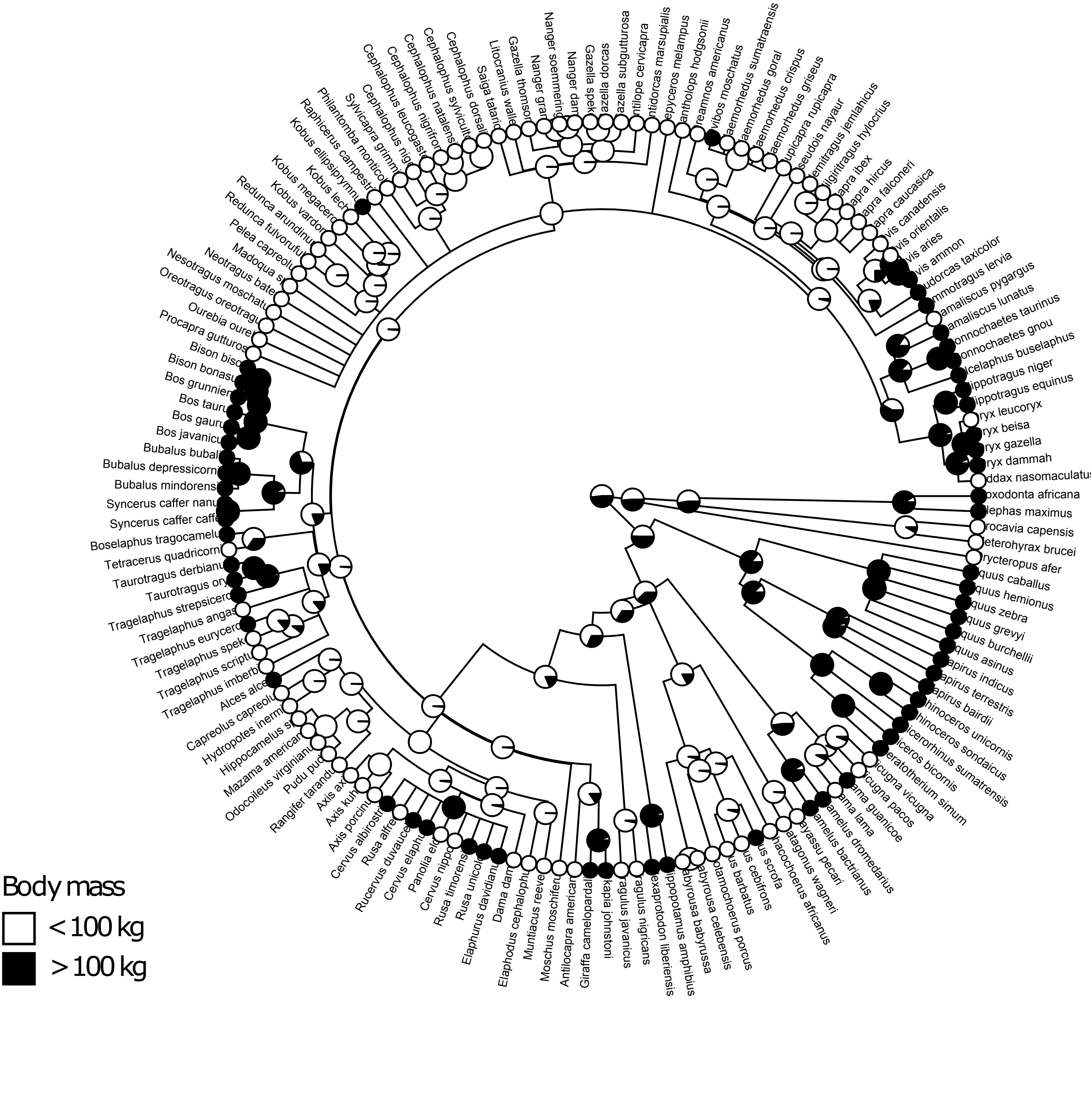

Fig. S4

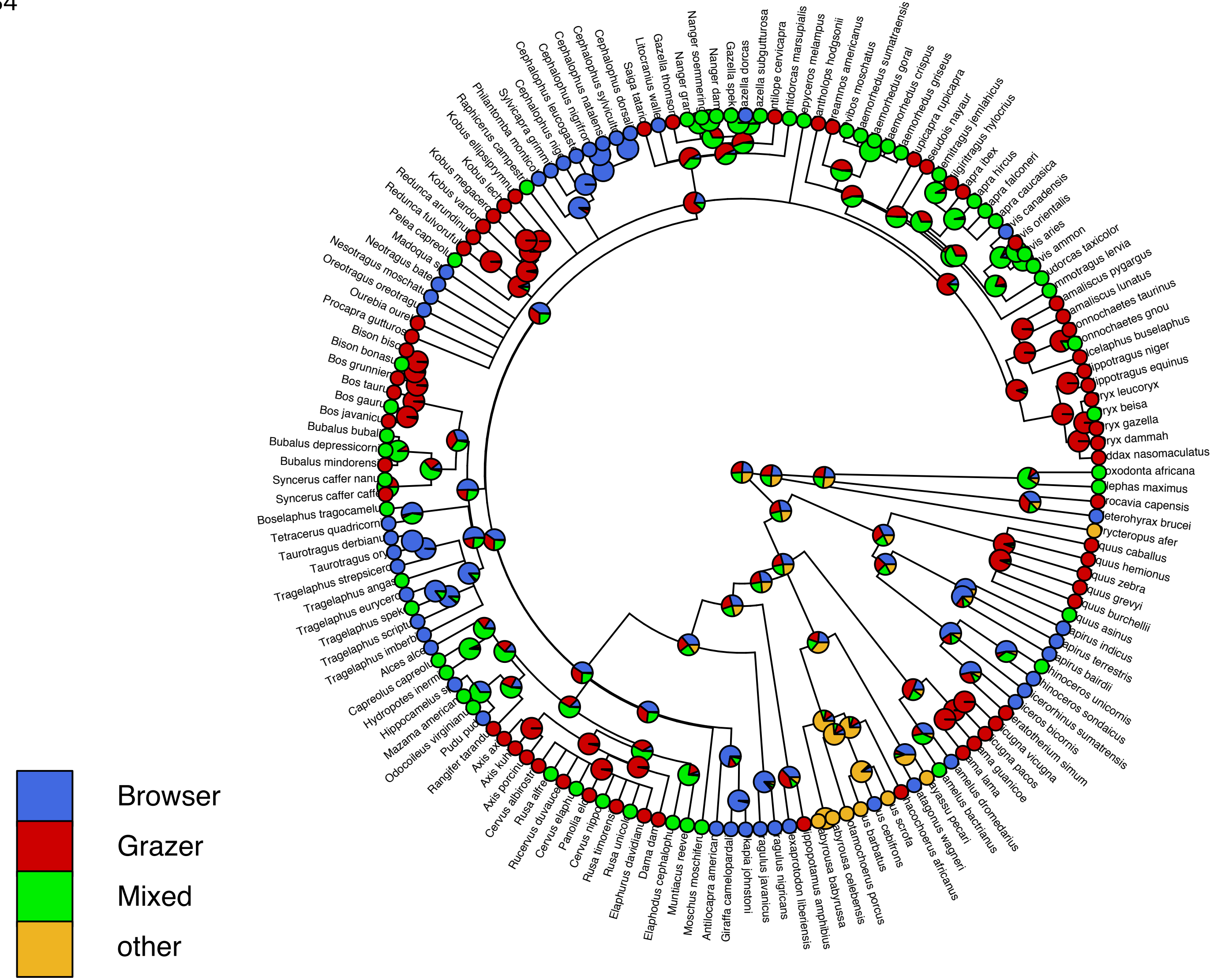

Browser
Grazer
Mixed
other
Cephalophus dorsali
Cephalophus sylvicult
Cephalophus natalensi
Cephalophus nigrifron
Cephalophus nig
Cephalophus leucogast
Sylvicapra grimm
Philantomba montico
Raphicerus campest
Kobus ellipsiprymnu
Kobus lech
Kobus megacero
Kobus vardon
Redunca arundinu
Redunca fulvorufu
Pelea capreol
Madoqua s
Neotragus bate
Nesotragus moschatu
Oreotragus oreotrag
Ourebia oure
Procapra gutturos
Bison biso
Bison bonasu
Bos grunnien
Bos tauru
Bos gauru
Bos javanicu
Bubalus bubal
Bubalus depressicorn
Bubalus mindorens
Syncerus caffer nanu
Syncerus caffer caffe
Boselaphus tragocamelu
Tetracerus quadricorn
Taurotragus derbianu
Taurotragus ory
Tragelaphus strepsicero
Tragelaphus angas
Tragelaphus eurycero
Tragelaphus spek
Tragelaphus script
Tragelaphus imberb
Alces alce
Capreolus capreol
Hydropotes inerm
Mazama american
Odocoileus virginianu
Pudu pud
Rangifer tarand
Axis ax
Axis kuh
Axis porcin
Cervus albirost
Rusa alfre
Rucervus duvauce
Cervus elaphu
Panolia eld
Cervus nippo
Rusa timorens
Rusa unicol
Elaphurus davidian
Dama dam
Elaphodus cephalophu
Muntiacus reeve
Moschus moschiferu
Antilocapra american
Giraffa camelopardal
kapia johnstoni
ragulus javanicus
ragulus nigricans
exaprotodon liberiensis
ippopotamus amphibius
abyrousa babyrussa
abyrousa celebensis
otamochoerus porcus
us barbatus
us cebifrons
us scrofa
hacochoerus africanus
atagonus wagneri
ayassu pecari
amelus bactrianus
amelus dromedarius
ama lama
ama guanicoe
icugna pacos
icugna vicugna
eratotherium simum
iceros bicornis
icerorhinus sumatrensis
hinoceros sondaicus
hinoceros unicornis
apirus bairdii
apirus terrestris
apirus indicus
quus asinus
quus burchellii
quus grevyi
quus zebra
quus hemionus
quus caballus
rycteropus afer
eterohyrax brucei
rocavia capensis
lephas maximus
oxodonta africana
ddax nasomaculatus
ryx dammah
ryx gazella
ryx beisa
ryx leucoryx
ippotragus equinus
ippotragus niger
lcelaphus buselaphus
onnochaetes gnou
onnochaetes taurinus
amaliscus lunatus
amaliscus pygargus
mmotragus lervia
udorcas taxicolor
vis ammon
vis aries
vis orientalis
vis canadensis
apra caucasica
apra falconeri
apra hircus
apra ibex
ilgiritragus hylocrius
emitragus jemlahicus
seudois nayaur
upicapra rupicapra
aemorhedus griseus
aemorhedus crispus
aemorhedus goral
aemorhedus sumatraensis
vibos moschatus
reamnos americanus
antholops hodgsonii
epyceros melampus
ntidorcas marsupialis
ntilope cervicapra
azella subgutturosa
azella dorcas
Gazella spek
Nanger dam
Nanger soemmering
Nanger gran
Gazella thomso
Litocranius walle
Saiga tataric

Fig. S5

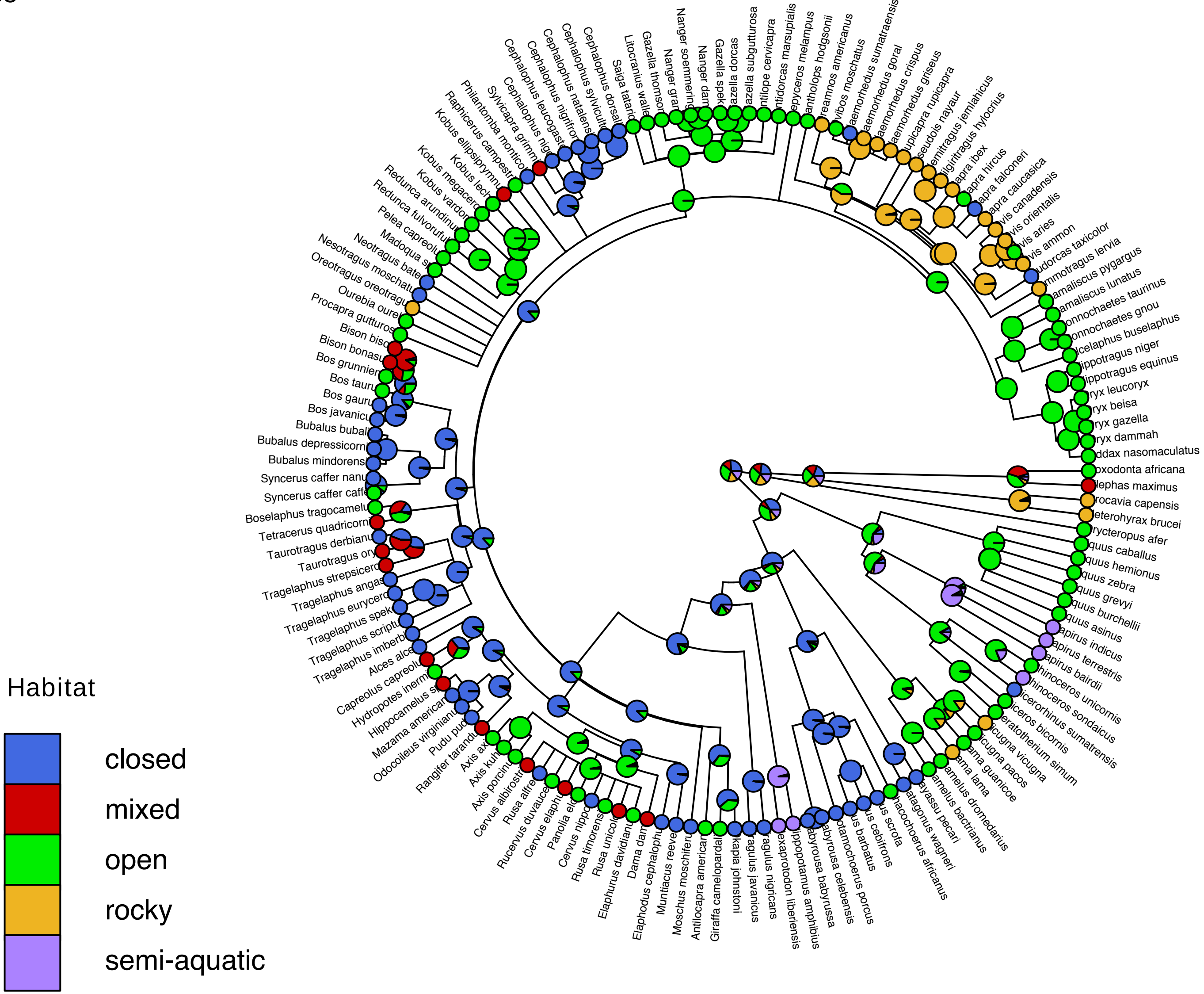

Fig. S6

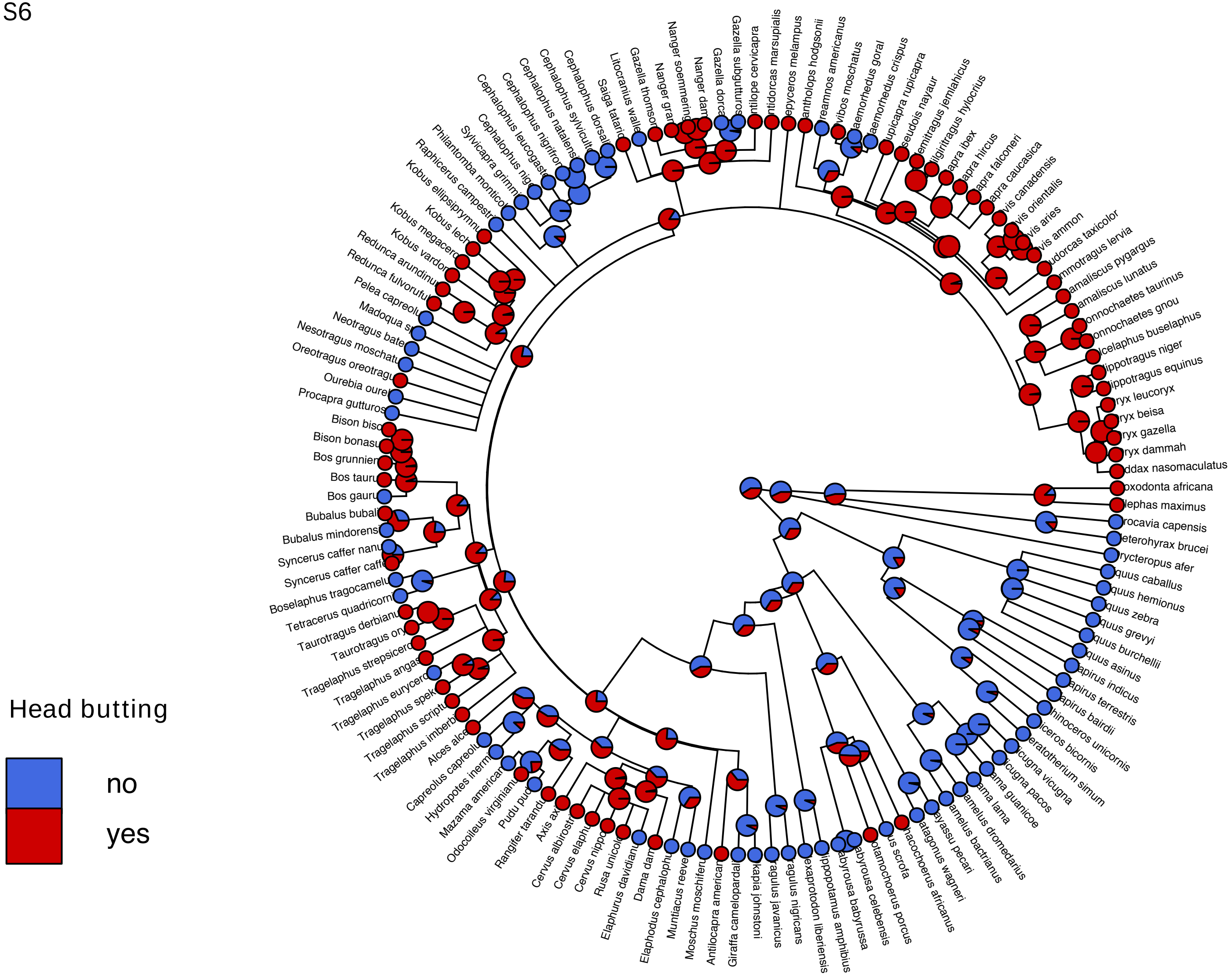

Head butting
no
yes
Loxodonta africana
Elephas maximus
Procavia capensis
Heterohyrax brucei
Orycteropus afer
Nanger soemmering
Nanger dam
Gazella dorca
Gazella subgutturosa
Antilope cervicapra
Antidorcas marsupialis
Aepyceros melampus
Pantholops hodgsonii
Oreamnos americanus
Ovibos moschatus
Naemorhedus goral
Naemorhedus crispus
Rupicapra rupicapra
Pseudois nayaur
Hemitragus jemlahicus
Nilgiritragus hylocrius
Capra ibex
Capra hircus
Capra falconeri
Capra caucasica
Ovis canadensis
Ovis orientalis
Ovis aries
Ovis ammon
Budorcas taxicolor
Ammotragus lervia
Damaliscus pygargus
Damaliscus lunatus
Connochaetes taurinus
Connochaetes gnou
Alcelaphus buselaphus
Hippotragus niger
Hippotragus equinus
Oryx leucoryx
Oryx beisa
Oryx gazella
Oryx dammah
Addax nasomaculatus
Litocranius walle
Gazella thomso
Nanger gran
Saiga tatarica
Cephalophus dorsali
Cephalophus sylvicultor
Cephalophus natalensis
Cephalophus nigrifron
Cephalophus leucogaster
Cephalophus nige
Sylvicapra grimmi
Philantomba montico
Raphicerus campestri
Kobus ellipsiprymnu
Kobus leche
Kobus megaceros
Kobus vardon
Redunca arundinu
Redunca fulvorufu
Pelea capreolu
Madoqua s
Neotragus bate
Nesotragus moschatu
Oreotragus oreotragu
Ourebia oure
Procapra gutturos
Bison biso
Bison bonasu
Bos grunnien
Bos tauru
Bos gauru
Bubalus bubali
Bubalus mindorensi
Syncerus caffer nanu
Syncerus caffer caffe
Boselaphus tragocamelu
Tetracerus quadricorni
Taurotragus derbianu
Taurotragus ory
Tragelaphus strepsicero
Tragelaphus angas
Tragelaphus eurycero
Tragelaphus speke
Tragelaphus scriptu
Tragelaphus imberbi
Alces alce
Capreolus capreolu
Hydropotes inermi
Mazama american
Odocoileus virginianu
Pudu pud
Rangifer tarandu
Axis ax
Cervus albirostri
Cervus elaphu
Cervus nippo
Rusa unicolo
Elaphurus davidian
Dama dam
Elaphodus cephalophu
Muntiacus reeve
Moschus moschiferu
Antilocapra american
Giraffa camelopardal
Okapia johnstoni
Tragulus javanicus
Tragulus nigricans
Hexaprotodon liberiensis
Hippopotamus amphibius
Babyrousa babyrussa
Babyrousa celebensis
Potamochoerus porcus
Sus scrofa
Phacochoerus africanus
Catagonus wagneri
Tayassu pecari
Camelus bactrianus
Camelus dromedarius
Lama lama
Lama guanicoe
Vicugna pacos
Vicugna vicugna
Ceratotherium simum
Diceros bicornis
Rhinoceros unicornis
Tapirus bairdii
Tapirus terrestris
Tapirus indicus
Equus asinus
Equus burchellii
Equus grevyi
Equus zebra
Equus hemionus
Equus caballus
